# Trends in the Incidence of Type 1 Diabetes in European Children and Adolescents from 1994 to 2022: A Systematic Review and Meta-Analysis

**DOI:** 10.1155/2024/2338922

**Published:** 2024-05-27

**Authors:** Marta Carolina Ruiz-Grao, Ana Díez-Fernández, Arthur E. Mesas, Vicente Martínez-Vizcaíno, Irene Sequí-Domínguez, Fernando Sebastián-Valles, Miriam Garrido-Miguel

**Affiliations:** ^1^ Facultad de Enfermería Universidad de Castilla-La Mancha 02006 AlbaceteSpain; ^2^ Health and Social Research Center Universidad de Castilla-La Mancha 16071 CuencaSpain; ^3^ Facultad de Enfermería Universidad de Castilla-La Mancha 16071 CuencaSpain; ^4^ Postgraduate Program in Public Health Universidade Estadual de Londrina 86057-970 Londrina ParanáBrazil; ^5^ Facultad de Ciencias de la Salud Universidad Autónoma de Chile 1101 TalcaChile; ^6^ Department of Endocrinology and Nutrition Instituto de Investigación Princesa Universidad Autónoma de Madrid Hospital Universitario de La Princesa MadridSpain

## Abstract

**Aim:**

To assess the incidence trends in type 1 diabetes among children and adolescents across Europe during the period from 1994 to 2022 using a systematic methodology.

**Materials and Methods:**

Cross-sectional or follow-up studies reporting population-based incidence rates (IRs) of European children and adolescents diagnosed aged <15 years with type 1 diabetes were included. The Mantel‒Haenszel or DerSimonian and Laird random-effects method was used to compute the pooled IR estimates and their 95% confidence intervals (CIs). Subgroup analyses were conducted by study year, biological sex, age group (0–4, 5−9, and 10–14 years), country, and European regions.

**Results:**

A total of 75 studies (219,331 children and adolescents aged 0–14 years) with data from 32 countries were included. Generally, a high overall rate of increase in type 1 diabetes incidence has been shown in most European countries from 1994 to 2022 in both sexes, with an overall increase from 10.85 (95% CI, 9.62–12.07) per 100,000 person-years from 1994 to 2003 to 20.96 (95% CI, 19.26–22.66) per 100,000 person-years from 2013 to 2022.

**Conclusions:**

There are substantial between-country differences in the current levels and trends of IR in type 1 diabetes in European children and adolescents. Our data suggest a worrying upward trend in most European countries.

## 1. Introduction

The incidence of newly diagnosed cases of type 1 diabetes worldwide is a concern for health systems, especially in children and adolescents [1]. In 2019, there were approximately 1.5 million individuals younger than 20 years worldwide with type 1 diabetes [1] and 16,300 global deaths due to diabetes (types 1 and 2 combined) occurred in people younger than 25 years and 73.7% (68.3–77.4) were classified as due to type 1 diabetes [2].

Exposure to different risk factors [3, 4, 5] in addition to genetic factors could have different influences depending on the country studied, especially exogenous exposures (e.g., environmental risk factors), and may be responsible for changes in the trend and incidence of type 1 diabetes in children and adolescents around the world [6].

Data from the International Diabetes Federation estimated that around 108,300 children and adolescents under 15 years would be diagnosed with type 1 diabetes in 2021, and this number rises to 149,500 when the age range is extended to under 20 years [7, 8]; besides a recent systematic review and meta-analysis showed that the incidence of type 1 diabetes in Europe from 1980 to 2019 was 15 per 100,000 individuals in the general population [9]. The 2019 report of the Europe and Diabetes Study (EURODIAB), which analyzed the trends of 22 European countries, including data from 1989 to 2013, indicated an overall pooled rate of annual increase of 3.4% (95% CI, 2.8%−3.9%) [10]. Europe is one of the continents where the data seem to be more accurate due to the EURODIAB registry [7, 8].

However, specific data from different studies are not included in the EURODIAB studies, such as incidence studies conducted in other regions [11, 12] or other centers in countries that are included in the EURODIAB Family Study.

Previous studies have explored the incidence of type 1 diabetes among children and adolescents under 15 years of age during different study periods [13, 14] or among individuals below 20 years old in a similar study period [15]. However, to date, no study has examined data on the incidence of type 1 diabetes in children and adolescents in the most European countries and regions with available data during the last three decades. Taking into account during this period, the COVID-19 pandemic phenomenon occurred, with the potential impact on the trend of type 1 diabetes in this population. Monitoring type 1 diabetes incidence trends across most European countries using the latest objectively measured data can also provide a more complete picture of the epidemiological situation in Europe and may help elucidate disparities across the continent. Therefore, the present systematic review and meta-analysis aimed to assess the incidence and trends in type 1 diabetes among European children and adolescents aged 0–14 years in Europe from 1994 to 2022.

## 2. Materials and Methods

**Figure 1:**
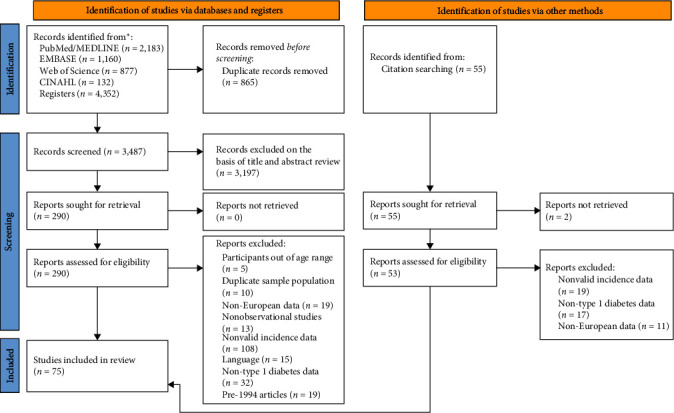
PRISMA 2020 flow diagram for new systematic reviews which included searches of databases and registers only.

This systematic review and meta-analysis were conducted according to the Preferred Reporting Items for Systematic Review and Meta-Analysis Protocols (PRISMA) (Figure 1) [16] and the Cochrane Collaboration Handbook [17]. We have registered and published this review on PROSPERO (registration number: CRD42021239480), and its protocol has been published elsewhere [18].

### 2.1. Search Strategy

We systematically searched the MEDLINE (via PubMed), Embase (via Scopus), CINAHL, and Web of Science databases for papers published from 1994 to December 2023. The following terms were combined to design the search strategy: (1) population (*child*, *children*, *childhood*, *schooler*, *toddlers*, *preadolescents*, *adolescent*, *infant*, *pediatr* ^*∗*^, *child* ^*∗*^, *teenag* ^*∗*^, *youth*, *young*, *school*, *school-aged*, *school-aged*); (2) outcome (*diabetes mellitus*, *diabetes mellitus type 1*, *diabetes mellitus*, *insulin-resistant*, *diabetes mellitus*, *insulin dependent*, *T1D*); (3) study design (*incidence*, *trend*, *epidemic* ^*∗*^, *observational*, *cross-sectional*, *longitudinal*, *survey*); and (4) location (*including terms for different European countries*). Medical Subject Headings of the National Library of Medicine and free terms were used to perform the MEDLINE search (Table S1).

The literature search, data extraction, and quality assessment were performed independently by two investigators (MG-M and MCR-G), and disagreements were resolved by consensus or involving a third researcher (AD-F).

### 2.2. Study Selection

The inclusion criteria were as follows: (1) studies reporting the population-based incidence rates (IR) of European children and adolescents aged <15 years diagnosed with type 1 diabetes; (2) observational studies (cross-sectional or follow-up studies (cohort studies)); (3) studies reporting data by year or period; and (4) studies including IR data.

Studies were excluded from the analyses when (1) they were published in languages other than English, Spanish, Italian, or Portuguese; (2) they did not provide details of the sampling method or sample composition; (3) the target population was a specific subgroup; or (4) they were duplicate reports of the same study. When more than one article provided data on the same sample, the article reporting the most detailed results and/or with the largest sample size was retained for data synthesis.

### 2.3. Search and Data Extraction

The main characteristics of the selected studies are summarized in Table S2 in the supplementary section, including information regarding (1) first author's name and publication year; (2) country; (3) European region; (4) level of representativeness (national/regional data); (5) period of study; (6) study design; (7) characteristics of the included population (age of participants and sample size); and (8) outcomes (mean annual incidence rates of type 1 diabetes by age group and total).

### 2.4. Quality Assessment

We used the Quality Assessment Tool for Observational Cohorts and Cross-Sectional Studies from the National Heart, Lung, and Blood Institute (NHLBI Cohort and Cross-sectional Studies). This tool evaluates the risk of bias according to 14 criteria: research question, study population, groups recruited from the same population and uniform eligibility criteria, sample size, justification, exposure assessed prior to outcome measurement, sufficient time frame to see an effect, different levels of exposure of interest, exposure measures and assessment, repeated exposure assessment, outcome measures, blinding of outcome assessors, follow-up rate, and statistical analyses. These criteria could be assessed as “yes,” “no,” or “other” (cannot be determined, not applicable, or not reported) [19].

Disagreements in the assessment of the risk of bias were discussed to reach a consensus (AD-F and MCR-G). A third researcher (MG-M) was consulted to come to the final decision when a consensus was not reached.

### 2.5. Statistical Analysis

The characteristics of the included studies are summarized in an ad hoc table. Data used to estimate the pooled incidence means were obtained from cross-sectional studies as well as from baseline measurements of longitudinal studies. The total incidence was categorized based on age group (0–4, 5−9, and 10–14.9 years old) and sex. Additionally, data were analyzed in different age groups, time periods (1994–2003, 2004−2012, 2013–2022), countries, and regions (Eastern, Northern, Southern, Western). Weighted pooling was used to study the size method in each analysis [20]. The effect size used was the IR per 100,000 person-years; for each country, the combined and stratified results by age group, sex, and period are presented. The Mantel‒Haenszel fixed-effects method [21] was used to compute the pooled incidence estimate and its 95% CI whenever there was no evidence of heterogeneity; otherwise, the DerSimonian and Laird random-effects method [22] was used. The heterogeneity of the results across studies was evaluated with the *I*^2^ statistic [23], which was interpreted as not important (0%–40%), moderate heterogeneity (30%–60%), substantial heterogeneity (50%–90%), or large heterogeneity (75%–100%). Considering the overlap between these heterogeneity categories, we decided to use the Mantel‒Haenszel fixed-effects method when *I*^2^ was less than 50% and the DerSimonian and Laird random-effects method when *I*^2^ was ≥50%.

In the subgroup analyses, we distinguished three time periods: 1994–2003, 2004−2012, and 2013–2022. We also distinguished four European regions: Eastern (Bosnia and Herzegovina, Croatia, Czech Republic, Hungary, Latvia, Lithuania, Montenegro, North Macedonia, Poland, Romania, Serbia, and Slovenia), Northern (Denmark, Estonia, Finland, Norway, and Sweden), Southern (Cyprus, Greece, Italy, Malta, Spain, and Turkey), and Western (Austria, Belgium, France, Germany, Ireland, Luxembourg, Switzerland, The Netherlands, and United Kingdom). Additionally, random-effects meta-regression analyses were used to evaluate whether the incidence estimate differed according to the gross national income per capita based on the purchasing power, parity of each European country, and latitude as an associated geographical factor.

The significance value of the pooled effect size was estimated based on the 95% CI. Two-sided *P* values of 0.05 or less were considered significant. Statistical analyses were performed using STATA V.15 software.

## 3. Results

### 3.1. Study Selection and Characteristics

The PRISMA diagram with the flow of studies through the review is presented in Figure 1. From the 4,187 articles identified, 75 (1.7%) met the inclusion criteria (Table S2) [10, 11, supplementary references (s)1-s73]. Of these, one study displayed data for several European countries (10). Studies were conducted in 32 European countries: Austria (3 reports), Belgium (1), Bosnia and Herzegovina (2), Croatia (3), Cyprus (2), Czech Republic (3), Denmark (1), Estonia (2), Finland (4), France (3), Germany (6), Greece (1), Hungary (2), Ireland (3), Italy (7), Latvia (1), Lithuania (3), Luxembourg (1), Malta (1), Montenegro (3), the Netherlands (2), North Macedonia (1), Norway (3), Poland (4), Romania (3), Serbia (2), Slovenia (2), Spain (16), Sweden (2), Switzerland (1), Turkey (3), and the United Kingdom (9).

A total of 219,331 children and adolescents aged 0–14 years were included in this review, with sample sizes of analyzed studies ranging from 41 to 30,840 participants with type 1 diabetes. In two studies [s17, s29], it was not possible to report the sample size. The reports were published between 1999 and 2023. The designs of the included studies were prospective [11, s1–s3, s6, s7, s9, s10, s12, s14, s16, s21, s27, s28, s31, s34, s36, s40, s49, s50, s52, s55, s57, s58, s60, s62, s63, s66, s70, s73] and prospective based on the capture–recapture method [s8, s19, s22, s23, s25, s29, s33, s43–s45, s48, s51, s56, s59] in 43 studies and retrospective in 15 studies [(s5, s7, s24, s30, s37–s39, s46, s53, s54, s64, s68, s69, s71, s72], and the rest of the studies were based on a mixed prospective–retrospective and capture–recapture method [10, s4, s11, s13, s15, s17, s18, s20, s32, s35, s41, s42, s47, s61, s65, s67]. Furthermore, 35 studies were based on national samples/registries [10, 11, s2, s5–s14, s17, s22, s23, s25–s33, s37, s38, s52, s53, s59, s60, s63, s65, s68, s70], 39 studies were based on regional samples [s1, s3, s4, s15, s16, s18, s19, s21, s24, s34, s35, s36, s39, s40–s51, s54, s58, s61, s62, s64, s66, s67, s69, s71–s73], and only one was based on national and regional samples [s20].

### 3.2. Study Quality

We evaluated 75 studies (100%) with a low risk of bias by the Quality Assessment Tool for Observational Cohort and Cross-Sectional Studies from the NHLBI. None of the included studies showed poor quality according to this scale. Specifically, the items that could not be assessed and were not taken into account for quality assessment using this scale were the following: *exposure assessed prior to outcome measurement* criteria (Item 6), *different levels of exposure of interest* criteria (Item 8), *exposure measures and assessment* criteria (Item 9), *repeated exposure assessment* criteria (Item 10), *blinding of outcome assessors* criteria (Item 12), and *the potential confounding variables measured and adjusted statistically for their impact on the relationship between exposure*(*s*) *and outcome*(*s*) (Item 14) (Table S3).

### 3.3. Incidence and Trends of Type 1 Diabetes Mellitus

Overall, the IR of type 1 diabetes in European children and adolescents aged 0–14 years increased from 10.85 (95% CI, 9.62–12.07) per 100,000 person-years from 1994 to 2003 to 20.96 (95% CI, 19.26–22.66) per 100,000 person-years from 2013 to 2022.

### 3.4. Time Trends by Country

Table 1 shows the IR of type 1 diabetes in children and adolescents (aged 0–14 years) by sex in 32 European countries from 1994 to 2022. From 1994 to 2003, the lowest IRs of type 1 diabetes were observed in Bosnia and Herzegovina (4.68; 95% CI, 1.84–7.52) and North Macedonia (4.95; 95% CI, 4.27–5.63), and the highest IRs were in Finland (39.55; 95% CI, 38.44–40.65) and Sweden (31.84; 95% CI, 29.29–34.48). From 2004 to 2012, the lowest IRs of type 1 diabetes were observed in Turkey (7.20; 95% CI, 5.00–9.40) and North Macedonia (7.45; 95% CI, 6.62–8.28), and the highest IRs were in Finland (55.75; 95% CI, 53.20–58.29) and Sweden (41.10; 95% CI, 36.76–45.45). Finally, from 2013 to 2022, data were available from 14 European countries. Thus, the lowest IRs of type 1 diabetes were observed in Romania (10.51; 95% CI, 9.58–11.55) and Turkey (12.77; 95% CI, 5.22–20.33); the highest IRs were in Finland (56.42; 95% CI, 47.80–65.04) and Ireland (32.63; 95% CI, 27.13–38.12). In most analyzed European countries, there is a significant increase in the IR of type 1 diabetes from 1994 to 2022. However, in countries such as the United Kingdom and Spain, there is some stabilization and even a slight decrease in the IR of type 1 diabetes from 2004 to 2022.

**Figure 2:**
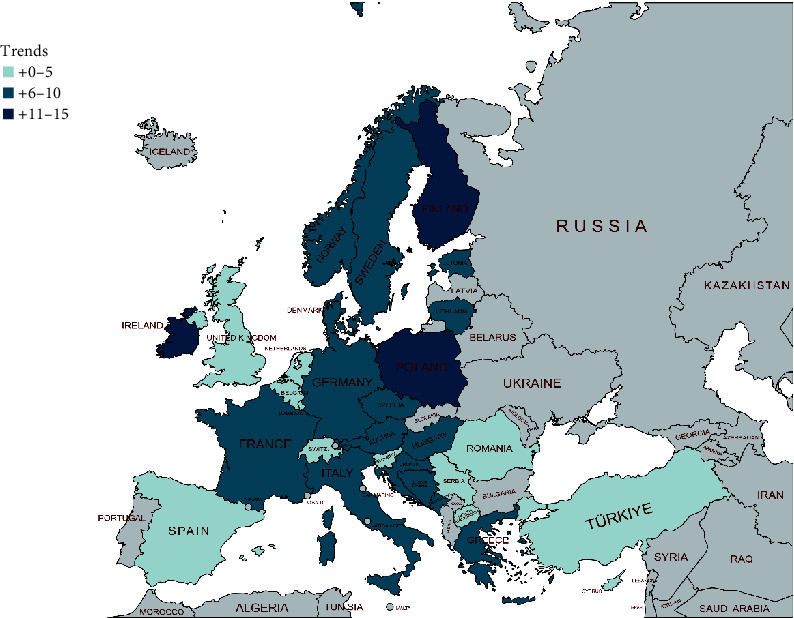
Spatial distribution of incidence rate changes in type 1 diabetes in children and adolescents from 1994 to 2022.

The analyzed spatial distribution of IR changes in type 1 diabetes in children and adolescents in different European countries is shown in Figure 2.

Table S4 provides sex-specific IR trends in each group (aged 0–4, 5−9, and 10–14 years) and country. In most European countries, boys presented a slightly higher IR of type 1 diabetes than girls, although these differences were not significant. However, significant differences were observed between the youngest group studied (0–4 years) and the oldest group studied (10–14 years) for both sexes.

### 3.5. Time Trends by Region

**Figure 3:**
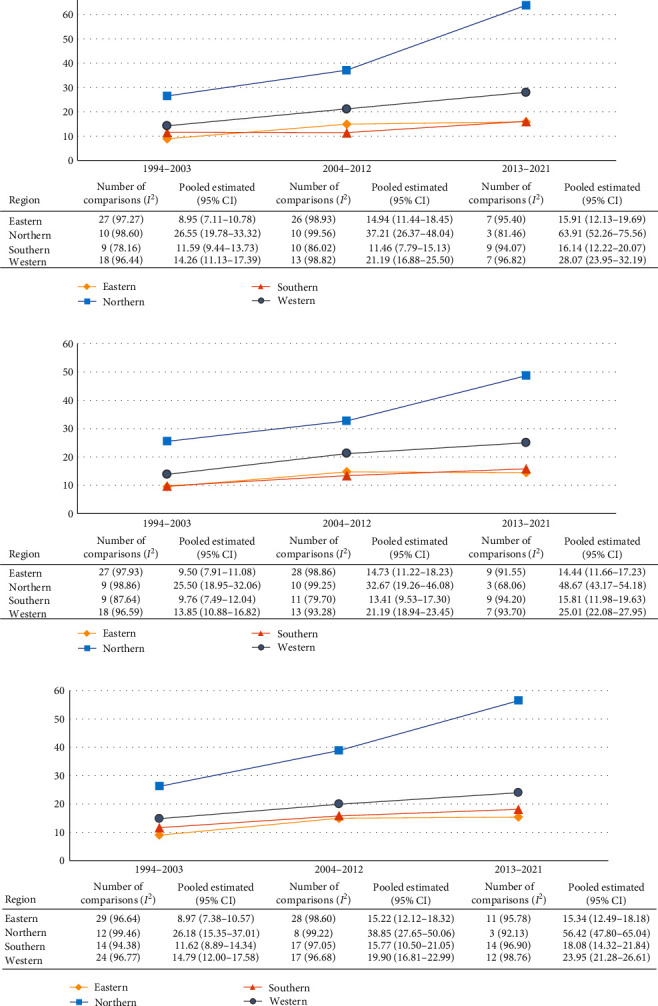
Pooled estimated (95% CI) and trends in the incidence rates of childhood type 1 diabetes (0–14 years) by sex across European regions. (a) Boys (aged 0–14 years), (b) girls (aged 0–14 years), and (c) overall (aged 0–14 years).

Figure 3 displays trends in the pooled IR estimates of type 1 diabetes in children (aged 0–14 years) from four regions of Europe stratified into three time periods (1994–2003, 2004–2012, and 2013–2022). Overall, from 1994 to 2022, the IR of type 1 diabetes increased in the Northern region from 26.18 (95% CI, 15.35–37.01) to 56.42 (95% CI 47.80–65.04) and also showed a significant increase in Eastern, Southern and Western Europe, where the type 1 diabetes incidence increased from 8.97 (7.38–10.57) to 15.34 (12.49–18.18), from 11.62 (8.89–14.34) to 18.08 (14.32–21.84), and from 14.79 (12.00–17.58) to 23.95 (21.28–26.61), respectively. The results for boys and girls are also shown in Figure 3. A significant increase in the trend is observed in several countries in the Northern European region (Finland, Sweden, or Norway), and for other regions, an increasing but not significant trend is observed. The country-specific increase in the IR of type 1 diabetes by sex for children and adolescents aged 0–14 years is shown in Table S4.

### 3.6. Meta-Regression

**Figure 4:**
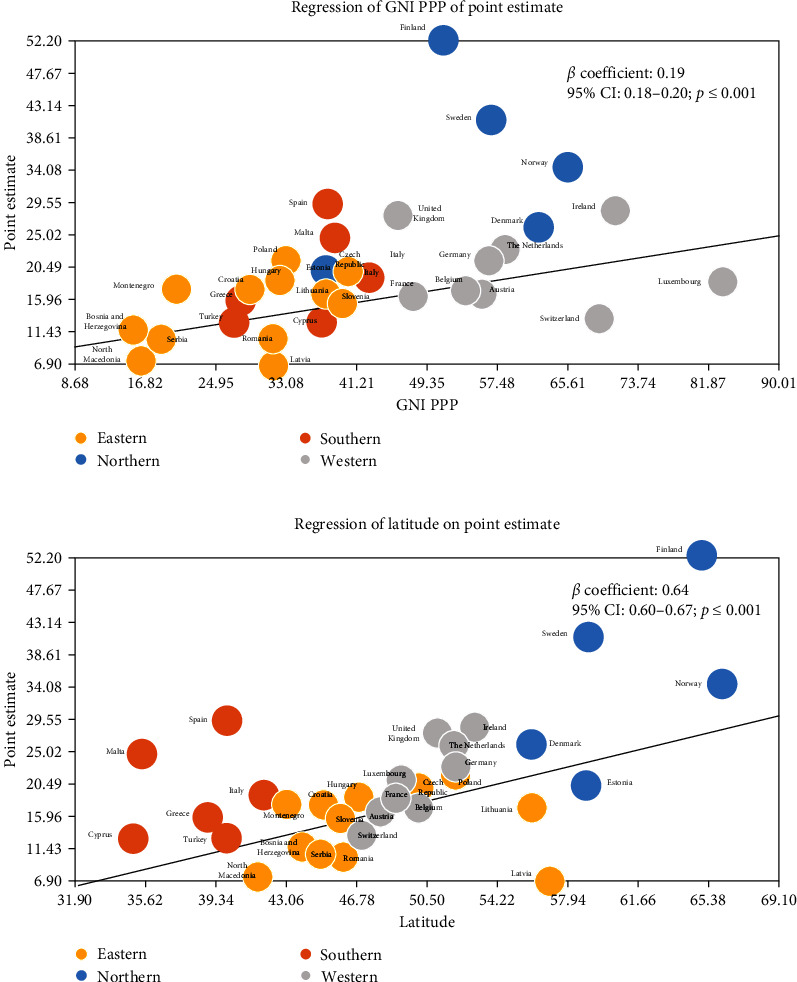
Meta-regression models. GNI PPP: gross national income per capita. (a) Incidence of type 1 diabetes and gross national income per capita in European countries and (b) Incidence of type 1 diabetes and geographical latitude in European countries.

Random-effects meta-regression models showed a positively significant association between the IR of type 1 diabetes and gross national income per capita in European countries (*ß* = 0.19, 95% CI, 0.18–0.20) (*p*  < 0.001) and latitude as a geographical factor (*ß* = 0.64, (95% CI, 0.60–0.67) (*p*  < 0.001)) (Figure 4).

## 4. Discussion

This systematic review and meta-analysis provide a comprehensive picture of the trends from 1994 to 2022 in the IR of type 1 diabetes in children and adolescents aged 0–14 years in 32 European countries. Overall, our results indicate a significant increase in the IR of type 1 diabetes in Europe from 10.85 per 100,000 person-years from 1994 to 2003 to 20.96 per 100,000 person-years during the period 2013–2022. Substantial differences between countries in both current levels and trends were found. In particular, some Northern countries (Finland, Sweden, or Norway) showed the highest incidence estimates, and Southern and Eastern countries had the lowest estimates. Finally, our data reveal an upward trend that has continued over the last years.

Different reasons could help to explain these changes in trends over the last two decades. First, the lower incidence rates reported during the first period could be related to an underreporting of type 1 diabetes cases. In addition, the increase in type 1 diabetes incidence during the last period may be attributed to improvements in the screening, diagnosis, and notification of true type 1 diabetes cases due to prevention or national programs [24]. Nevertheless, as incidence rates continue to increase in some European countries, especially in countries belonging to the Northern European region, not only genetic susceptibility [6] but also other socioeconomic, environmental, geographic, or lifestyle risk factors may be behind the marked differences in type 1 diabetes incidence [3, 25, 26].

In this regard, it has already been shown that a high body mass index (BMI) can exacerbate islet autoimmunity before clinical type 1 diabetes, particularly in children at lower risk based on age and human leukocyte antigen (HLA) levels [27]. Moreover, it has been observed that the increase in BMI might explain, at least in part, why minority ethnic groups present type 1 diabetes with a worse prognosis [28]. Thus, the increase in overweight and obesity in children could be a determining factor in the recent increase in the incidence of type 1 diabetes.

Socioeconomic status (SES) could be associated with type 1 diabetes. In countries/continents with high gross domestic product (GDP), type 1 diabetes incidence is higher than in countries/continents with low GDP [29]. This could be explained by an improvement in the diagnosis procedures in high GDP (Europe and North America); a decrease in type 1 diabetes incidence was observed in continents with low GDP and the largest child populations (West Indies), where the mortality in children is high before 5 years old due to infectious diseases [30].

Our study showed a significant positive association between the incidence of type 1 diabetes and gross national income per capita in European countries, which is in line with the findings of previous studies [31]. Similarly, another article that examined neighborhood-level risk factors for type 1 diabetes in the United States showed that higher levels of SES were associated with a higher risk of type 1 diabetes, and the proportion of crowded households or poverty was associated with a low risk of type 1 diabetes [31]. A phenomenon to highlight is the significant increase in autoimmune diseases taking place while the incidence of infectious diseases decreased due to vaccination programs, better hygiene conditions, or the use of antibiotics [4].

Geographical and environmental risk factors could also be associated with type 1 diabetes. Some studies have already evaluated the hypothesis of possible migratory phenomena and have shown that the offspring of the transmigratory population had a rising incidence of childhood diabetes, which was approaching that of the indigenous population [32]. Although, in Sweden, where the increase in the incidence of type 1 diabetes in the late twentieth century has been approaching a more stable, albeit high level over the last two decades, the increased immigration from countries with lower incidences of type 1 diabetes did not provide a complete explanation for an observed leveling off [33]. Another geographical risk factor may be latitude because some studies have suggested a positive association between the latitude north of the equator and the risk of type 1 diabetes. This risk increased on average from 3.3% to 3.5% for each degree of latitude away from the equator [33]. These results could be in line with our findings in meta-regression; we have also shown that in some European countries, such as Norway, Sweden, or Finland, the incidence of type 1 diabetes is higher than that in other countries further south. This hypothesis could be explained by the fact that the exposure to ultraviolet radiation (UVR) reaching the Earth's surface varies inversely with latitude and is a prominent latitude-related environmental factor.

Some studies have shown that UVR downregulates cellular immunity, attenuating T-helper (Th)1 T-cell–mediated immune responses [34]. These responses are considered involved in some autoimmune disorders, such as type 1 diabetes. One study found an inverse association between UVR levels in Australia and type 1 diabetes prevalence and incidence [35]. It may be by the fact that UVR exposure could be protective against Th1-mediated disorders such as type 1 diabetes by downregulating Th1 autoimmune responses by several different immunoregulatory mechanisms. One of these mechanisms involves UVR-induced vitamin D.

The protective role of UVR in type 1 diabetes may act through its important role in vitamin D synthesis in the skin. Similarly, a decreased incidence of type 1 diabetes was shown in children supplemented with vitamin D in infancy [36]. One study showed that maternal intake of vitamin D during pregnancy and high doses of vitamin D early in life were protective factors against autoimmunity and type 1 diabetes [36].

Moreover, vitamin D receptor (VDR) polymorphisms have been investigated in type 1 diabetes, and some studies have reported that individual VDR polymorphisms seem not to be associated with type 1 diabetes risk [5]. However, haplotypes could contribute significantly to disease susceptibility, and it is suggested that in type 1 diabetes pathogenesis, VDR polymorphisms interact with each other and with environmental factors, such as the previously mentioned latitude and microbial environment [5].

However, the north–south gradient in Europe, which has been evidenced in some studies [37], is not observed in the case of countries such as Spain, which is at a lower latitude but where the incidence is high. In this case, it could be environmental factors that are intense enough to trigger autoimmune processes and give rise to type 1 diabetes in childhood, including perinatal infections and the type of feeding, including breastfeeding, rapid growth, or weight evolution, among others [6].

Additionally, the role of the gut microbiome is a recent risk factor that could be related to type 1 diabetes [6]. On the other hand, the role of breastfeeding in the risk of T1D remains unclear, and the consequences of initiating or prolonging breastfeeding are still controversial [38]. The Mediterranean diet and the consumption of dietary fiber and/or probiotics are being studied as possible modifiers of the onset and progression of this disease by their role modulators of the intestinal microflora and the reduction of the associated proinflammatory profile relationship to type 1 diabetes [39].

Other prominent environmental factors related to type 1 diabetes incidence in Europe could be the increasing exposure of the population to various food chemicals, as well as to viral infections because of increased mobility. Seasonality is another risk factor studied [6], with a peak of the incidence of type 1 diabetes registered in the cold season that could be explained by the involvement of seasonal viral infections [40]. It is known that viruses are a common environmental risk factor and that early exposure to microbes and other pathogens early in life promotes immune responses and protects against the risk of autoimmunity in diseases such as type 1 diabetes [41]. In this sense, exposure to a persistent cold climate over time could explain why the incidence of type 1 diabetes is higher in countries belonging to the Northern European region.

On the other hand, it is possible to observe the presence of the phenomenon of the COVID-19 pandemic and its effect on chronic pathologies during the last studied period (2013–2022). Our findings are similar to those of another recent meta-analysis that showed an increased incidence rates of type 1 diabetes in children and adolescents during vs. before the COVID-19 pandemic around the world. This annual increase in the incidence was greater than the 3%–4% expected, based on prepandemic temporal trends in Europe [42].

Exploration of potential mechanisms linking SARS-CoV-2 infection to new-onset diabetes includes immune-mediated responses, dysregulated glucose metabolism, and direct viral damage to *β* cells, thereby hindering compensatory mechanisms and leading to *β*-cell exhaustion. Additionally, lifestyle changes, shifts in pediatric non-COVID-19 infection patterns, increased stress, and social isolation are considered potential contributors. However, all of these mechanisms are unclear [42]. Furthermore, Rahmati et al. [43] reported a noteworthy increase in childhood new-onset type 1 diabetes incidence during the first year of the COVID-19 pandemic (32.39 per 100,000 children) compared to the pre-COVID-19 period (19.73 per 100,000 children) worldwide, as revealed by a systematic review and meta-analysis.

Finally, in both children and adolescents, our study showed a higher incidence in boys than girls in most European countries, with no statistically significant differences, especially in high-incidence countries. One study showed that the annual rate of type 1 diabetes increased more in boys than in girls in the 10- to 14-year-old age group (3.3% and 2.6% per annum, respectively) in 26 European centers from 1989 to 2013 [8, 10]. This phenomenon has been observed in worldwide studies and in several countries [6]. Possible explanations for sex differences could be that the different effects of environmental risk factors and lifestyle on type 1 diabetes incidence in females and males during childhood onset may have different manifestations in the sexes [44]. Additionally, according to our results, the incidence of type 1 diabetes increases with age up to a peak at approximately 10–14 years, consistent with the findings of worldwide studies, even in low-incidence countries such as China [45].

## 5. Conclusions

In conclusion, this systematic review and meta-analysis shows that in most countries for which data are available, the IR of type 1 diabetes in children and adolescents has increased in recent decades. Our data suggest an upward trend in some European countries, such as those belonging to the northern region, being a worrisome phenomenon. However, due to the more limited number of studies available, especially in the last period where the COVID-19 pandemic phenomenon was present, our results should be interpreted with caution. Additionally, an interaction between genetic, behavioral, and environmental risk factors and type 1 diabetes has been suggested in this population. Further studies are needed to clarify this interaction. In the meantime, policies to promote healthy behaviors and to control environmental factors that could influence the incidence of some health disorders related to immunity, such as type 1 diabetes, could be of great interest.

## Figures and Tables

**Table 1:** Trends in the incidence rates (pool estimate (95% CI) of childhood diabetes type 1 (0–14 years old) by sex for 32 countries of Europe.

Country	1994–2003	2004–2012	2013–2022	*Δ* diabetes 1994–2022
Austria				
Boys	10.88 (9.28–12.48)^#^	17.24 (12.50–22.00)^#^	—	+6.36^a^
Girls	10.22 (9.08–11.37)^#^	15.81 (12.87–18.75)^#^	—	+5.59^a^
Overall	10.53 (9.35–11.71)^#^	16.56 (12.74–20.38)^#^	—	+6.03^a^
Belgium				
Boys	16.01 (14.01–18.01) ^*∗*^	17.75 (15.75–19.75) ^*∗*^	—	+1.75
Girls	15.16 (13.16–17.16) ^*∗*^	16.63 (14.63–18.63) ^*∗*^	—	+1.47
Overall	14.87 (12.84–16.5) ^*∗*^	17.20 (15.23–19.17) ^*∗*^	—	+2.33
Bosnia and Herzegovina				
Boys	3.96 (2.57–5.34)^#^	9.50 (7.42–11.58) ^*∗*^	16.6 (13.9–19.4) ^*∗*^	+12.68^a,b,c^
Girls	4.30 (1.99–6.62)^#^	10.50 (8.29–12.71) ^*∗*^	14.79 (12.16–17.37) ^*∗*^	+10.49^a,b^
Overall	4.68 (1.84–7.52)^#^	12.10 (10.34–13.86) ^*∗*^	14.54 (12.83–16.26) ^*∗*^	+9.86^a,b^
Croatia				
Boys	11.70 (7.04–16.37)^#^	17.20 (16.01–18.40) ^*∗*^	—	+5.50
Girls	10.05 (7.05–13.04)^#^	19.32 (16.06–22.57)^#^	—	+9.27^a^
Overall	10.87 (7.06–14.68)^#^	17.57 (16.56–18.58) ^*∗*^	—	+6.70^a^
Cyprus				
Boys	11.20 (8.20–14.20) ^*∗*^	13.30 (9.90–16.70) ^*∗*^	—	+2.10
Girls	9.70 (6.90–12.50) ^*∗*^	16.50 (12.60–20.40) ^*∗*^	—	+6.80^a^
Overall	10.48 (8.40–12.56) ^*∗*^	12.89 (9.20–16.58)^#^	—	+2.41
Czech Republic				
Boys	12.71 (9.19–16.24)^#^	20.99 (19.69–22.23)^#^	—	+8.28^a^
Girls	13.03 (10.20–15.85)^#^	18.99 (17.59–20.40)^#^	—	+5.96^a^
Overall	12.83 (9.60–16.06)^#^	19.90 (18.58–21.22)^#^	—	+7.07^a^
Denmark				
Boys	18.71 (17.71–19.71) ^*∗*^	26.38 (25.38–27.38) ^*∗*^	—	+7.67^a^
Girls	20.13 (19.13–21.13) ^*∗*^	25.80 (24.80–26.80) ^*∗*^	—	+5.67^a^
Overall	19.40 (18.42–20.38) ^*∗*^	26.10 (24.48–27.72) ^*∗*^	—	+6.70^a^
Estonia				
Boys	13.71 (10.83–16.59)^#^	21.40 (16.35–26.45) ^*∗*^	—	+7.70
Girls	11.44 (9.96–12.93)^#^	20.20 (15.09–25.31) ^*∗*^	—	+8.80^a^
Overall	14.09 (8.75–19.44)^#^	—	—	—
Finland				
Boys	46.91 (35.20–58.62)^#^	61.12 (59.06–63.18) ^*∗*^	63.35 (51.70–75.01)^#^	+16.44^a^
Girls	43.76 (34.71–52.80)^#^	50.15 (48.27–52.04) ^*∗*^	48.67 (43.17–54.18)^#^	+4.91
Overall	39.55 (38.44–40.65) ^*∗*^	55.75 (53.20–58.29)^#^	56.42 (47.80–65.04)^#^	+16.87^a,b^
France				
Boys	7.06 (6.00–8.26) ^*∗*^	—	19.60 (18.50–20.70) ^*∗*^	+12.54^b^
Girls	7.13 (6.03–8.36) ^*∗*^	—	18.70 (17.5–19.8) ^*∗*^	+12.00^b^
Overall	10.78 (9.23–12.34)^#^	15.90 (14.92–16.88)^#^	17.56 (16.48–18.64)^#^	+6.78^a,b^
Germany				
Boys	17.42 (13.48–21.37)^#^	23.30 (22.72–23.87) ^*∗*^	28.37 (25.63–31.11)^#^	+10.95^a,b,c^
Girls	16.35 (12.94–19.77)^#^	21.63 (20.23–23.04)^#^	23.30 (22.38–24.22) ^*∗*^	+6.95^a,b^
Overall	16.22 (13.86–18.57)^#^	21.58 (19.80–23.36)^#^	25.30 (22.36–28.20)^#^	+9.08^a,b^
Greece				
Boys	8.89 (6.61–11.17) ^*∗*^	10.81 (8.52–13.10) ^*∗*^	16.50 (12.00–22.10) ^*∗*^	+7.61^b^
Girls	6.50 (4.56–8.44) ^*∗*^	10.05 (7.65–12.45) ^*∗*^	15.10 (10.90–20.50) ^*∗*^	+8.60^b^
Overall	7.56 (5.84–9.28) ^*∗*^	10.35 (8.52–12.19) ^*∗*^	15.80 (7.70–18.90) ^*∗*^	+8.24
Hungary				
Boys	11.40 (10.80–12.00) ^*∗*^	20.15 (19.55–20.75) ^*∗*^	—	+8.75^a^
Girls	11.66 (11.06–12.26) ^*∗*^	18.18 (17.58–18.78) ^*∗*^	—	+6.52^a^
Overall	11.50 (10.55–12.45)^#^	18.81 (18.18–19.44) ^*∗*^	—	+7.30^a^
Ireland				
Boys	16.80 (13.20–21.10) ^*∗*^	28.75 (27.31–30.19) ^*∗*^	33.52 (27.77–39.27)^#^	+16.72^a,b^
Girls	16.30 (12.60–20.70) ^*∗*^	26.36 (24.93–27.79) ^*∗*^	31.90 (27.02–36.78)^#^	+15.60^a,b^
Overall	16.60 (13.90–19.50)^#^	26.72 (24.45–28.99) ^*∗*^	32.63 (27.13–38.12)^#^	+16.03^a,b^
Italy				
Boys	13.08 (12.63–13.54) ^*∗*^	18.39 (4.32–32.45) ^*∗*^	21.39 (15.83–26.95)^#^	+8.31^b^
Girls	11.39 (10.96–11.83) ^*∗*^	18.60 (7.37–29.57) ^*∗*^	23.03 (20.26–25.78) ^*∗*^	+11.64^b^
Overall	12.26 (11.94–12.58) ^*∗*^	19.04 (17.85–20.23) ^*∗*^	21.50 (10.19–24.80)^#^	+9.24^a^
Latvia				
Boys	6.70 (5.70–7.70) ^*∗*^	—	—	—
Girls	7.20 (6.10–8.30) ^*∗*^	—	—	—
Overall	6.90 (6.13–7.67) ^*∗*^	—	—	—
Lithuania				
Boys	7.80 (6.61–8.99)^#^	16.51 (15.61–17.41) ^*∗*^	—	+8.70^a^
Girls	8.91 (7.53–10.28)^#^	17.61 (16.71–18.51) ^*∗*^	—	+9.00^a^
Overall	8.33 (7.11–9.55)^#^	17.05 (15.91–18.19) ^*∗*^	—	+8.72^a^
Luxembourg				
Boys	13.26 (10.46–16.06) ^*∗*^	19.51 (16.71–22.31) ^*∗*^	—	+6.24^a^
Girls	14.80 (12.00–17.60) ^*∗*^	17.48 (14.68–20.28) ^*∗*^	—	+2.68
Overall	14.00 (11.43–16.57) ^*∗*^	18.50 (15.67–21.33) ^*∗*^	—	+4.50
Malta				
Boys	—	—	—	—
Girls	—	—	—	—
Overall	—	24.68 (21.94–27.43) ^*∗*^	—	—
Montenegro				
Boys	13.46 (12.57–14.34) ^*∗*^	18.91 (16.90–20.93) ^*∗*^	—	+5.44^a^
Girls	12.30 (10.39–14.20) ^*∗*^	16.81 (14.91–18.71) ^*∗*^	—	+4.50^a^
Overall	11.94 (10.21–13.66) ^*∗*^	17.55 (15.60–19.49) ^*∗*^	20.55 (14.62–26.49) ^*∗*^	+8.61^a,b^
North Macedonia				
Boys	5.01 (4.21–5.81) ^*∗*^	7.21 (6.41–8.01) ^*∗*^	—	+2.20^a^
Girls	4.88 (4.08–5.68) ^*∗*^	7.13 (6.33–7.93) ^*∗*^	—	+2.25^a^
Overall	4.95 (4.27–5.63) ^*∗*^	7.45 (6.62–8.28) ^*∗*^	—	+2.50^a^
Norway				
Boys	28.46 (27.46–29.46) ^*∗*^	34.40 (33.54–35.27) ^*∗*^	—	+5.95^a^
Girls	26.61 (25.61–27.61) ^*∗*^	27.54 (20.05–35.04)^#^	—	+0.93
Overall	25.54 (22.09–28.99)^#^	32.70 (31.50–34.00) ^*∗*^	—	+7.16^a^
Poland				
Boys	10.43 (9.54–11.32) ^*∗*^	17.14 (14.77–19.52)^#^	23.15 (21.10–25.20) ^*∗*^	+12.73^a,b,c^
Girls	10.53 (10.43–10.63) ^*∗*^	15.01 (12.56–17.46)^#^	17.59 (14.85–20.34)^#^	+7.06^a,b^
Overall	10.42 (9.77–11.08) ^*∗*^	17.63 (15.72–19.53)^#^	21.49 (20.07–22.91) ^*∗*^	+11.07^a,b,c^
Romania				
Boys	5.54 (4.58–6.49)^#^	8.68 (7.53–9.82)^#^	10.85 (9.98–11.71) ^*∗*^	+5.30^a,b,c^
Girls	5.65 (5.04–6.27)^#^	8.04 (7.17–8.92)^#^	10.15 (9.30–10.99)^#^	+4.50^a,b,c^
Overall	5.60 (4.93–6.27)^#^	8.62 (7.66–9.57)^#^	10.51 (9.58–11.55)^#^	+4.90^a,b,c^
Serbia				
Boys	10.60 (9.50–11.80) ^*∗*^	—	—	—
Girls	16.40 (14.00–19.20) ^*∗*^	—	—	—
Overall	10.60 (9.80–11.40) ^*∗*^	—	13.68 (6.86–20.51)^#^	+3.08
Slovenia				
Boys	9.73 (8.63–10.83) ^*∗*^	13.98 (12.88–15.08) ^*∗*^	—	+4.25^a^
Girls	10.48 (9.38–11.58) ^*∗*^	16.11 (15.01–17.21) ^*∗*^	—	+5.63^a^
Overall	10.10 (9.00–11.20) ^*∗*^	15.60 (14.15–17.05) ^*∗*^	—	+5.50^a^
Spain				
Boys	12.74 (9.85–15.64)^#^	14.39 (12.47–16.31)^#^	13.09 (7.80–18.38)^#^	+0.35
Girls	11.13 (9.23–13.02)^#^	17.08 (13.42–20.74)^#^	11.36 (5.98–16.68)^#^	+0.23^a^
Overall	15.99 (14.43–17.54)^#^	20.83 (16.75–24.91)^#^	17.47 (10.35–24.52) ^*∗*^	+1.48
Sweden				
Boys	32.80 (30.03–35.57)^#^	42.41 (37.92–46.91)^#^	—	+9.60^a^
Girls	30.94 (28.15–33.74)^#^	39.25 (34.57–43.94)^#^	—	+8.31^a^
Overall	31.84 (29.29–34.38)^#^	41.10 (36.76–45.45)^#^	—	+9.26^a^
Switzerland				
Boys	10.23 (9.50–10.96) ^*∗*^	13.61 (12.90–14.32) ^*∗*^	—	+3.39^a^
Girls	9.00 (8.30–9.70) ^*∗*^	23.30 (22.66–23.90) ^*∗*^	—	+14.30^a^
Overall	9.65 (9.08–10.22) ^*∗*^	13.25 (12.58–13.92) ^*∗*^	—	+3.60^a^
The Netherlands				
Boys	—	20.80 (19.22–22.5) ^*∗*^	—	—
Girls	—	22.00 (20.40–23.80) ^*∗*^	—	—
Overall	18.10 (16.60–19.60) ^*∗*^	23.08 (19.66–26.51)^#^	—	+5.00^a^
Turkey				
Boys	—	5.70 (2.99–8.41) ^*∗*^	12.76 (5.10–20.41)^#^	+7.06
Girls	—	8.70 (5.22–12.18) ^*∗*^	12.61 (5.28–19.95)^#^	+3.90
Overall	—	7.20 (5.00–9.40) ^*∗*^	12.77 (5.22–20.33)^#^	+5.57
United Kingdom				
Boys	23.99 (21.35–26.62)^#^	29.47 (23.81–35.13)^#^	22.20 (20.30–24.10) ^*∗*^	−1.7
Girls	23.46 (20.95–25.96)^#^	28.21 (22.02–34.41)^#^	20.10 (18.30–21.90) ^*∗*^	−3.36^c^
Overall	23.18 (20.73–25.64)^#^	27.63 (23.64–31.61)^#^	21.30 (19.9–22.70) ^*∗*^	−1.88^c^

^a^Statistical significance between 1994 and 2012; ^b^statistical significance between 1994 and 2022; ^c^statistical significance between 2004 and 2022;  ^*∗*^Mantel‒Haenszel fixed-effects method; ^#^DerSimonian and Laird random-effects method.

## Data Availability

All data are available upon request to the corresponding author.

## Additional Points

*Highlights*. The incidence rate of type 1 diabetes in European children and adolescents aged 0–14 years increased from 10.85 per 100,000 person-years from 1994 to 2003 to 20.96 per 100,000 person-years from 2013 to 2022. Trends are increasing in most European countries, especially those belonging to the Northern region (such as Finland, Sweden, or Norway). However, in countries such as the United Kingdom and Spain, there is some stabilization and even a slight decrease during last years. Genetic, behavioral, and environmental risk factors related with type 1 diabetes need further research. *Limitations*. There were some limitations of this meta-analysis that should be acknowledged. Some of these are common to meta-analyses (e.g., selection bias and limited availability of complete information from study reports), but others are particularly important in our study. First, not all studies ensured representative samples of the population, which posed a threat to the validity of their estimates. Second, only studies that reported data by year or periods of time and included incidence rates were included, which limited the comparability with other relevant studies. Third, there were limited data during the 2013–2022 period, due to the low number of studies available in this period; thus, our findings for this period should be taken with caution. Fourth, for our analyses, because of the scarcity of follow-up studies involving the same people over time, the analysis of time series was discarded. Fifth, differences in sample characteristics, number of studies by country, limited number of national-based samples, geographic location, and the quality of the included data may have increased heterogeneity between studies, which may have reduced the quality of evidence on incidence and type 1 diabetes trends.

## Supplementary Materials

Supplementary Materials Table S1: search strategy for the MEDLINE database. Table S2: characteristics of studies included in the systematic review and or meta-analysis. Table S3: quality assessment (NHLBI cohort and cross-sectional studies). Table S4: trends in the incidence rates (pool estimate (95% confidence interval) of childhood diabetes type 1 by sex and age group for 32 countries of Europe). References of included studies and PRISMA checklist.
