# Supplementary material for: Trends in the Incidence of Type 1 Diabetes in European Children and Adolescents from 1994 to 2022: A Systematic Review and Meta-Analysis
Source: Pediatr Diabetes. 2024 May 27;2024:2338922. doi: 10.1155/2024/2338922 (PMC12020782; doi:10.1155/2024/2338922)
Supplement: Supplementary Materials — Table S1: search strategy for the MEDLINE database. Table S2: characteristics of studies included in the systematic review and or meta-analysis. Table S3: quality assessment (NHLBI cohort and cross-sectional studies). Table S4: trends in the incidence rates (pool estimate (95% confidence interval) of childhood diabetes type 1 by sex and age group for 32 countries of Europe). References of included studies and PRISMA checklist. [file 2338922.f1.docx]

**Supplementary Material**

[Supplementary Table 1. Search strategy for the MEDLINE database 2](#_heading=h.gjdgxs)

[Supplementary Table 2. Characteristics of studies included in the systematic review and or meta-analysis 4](#_heading=h.30j0zll)

[Supplementary Table 3. Quality Assessment (NHLBI Cohort and Cross-sectional Studies) 34](#_heading=h.1fob9te)

[Supplementary Table 4. Trends in the incidence rates (pool estimate (95% confidence interval) of childhood diabetes type 1 by sex and age group for 32 countries of Europe 37](#_heading=h.3znysh7)

[References of included studies 46](#_heading=h.2et92p0)

[PRISMA Checklist 51](#_heading=h.tyjcwt)

# Supplementary Table 1. Search strategy for the MEDLINE database

|  | |
| --- | --- |
| **Search Set Medline** | |
| #1 | Children [All Fields] |
| #2 | Childhood [All Fields] |
| #3 | Schooler [All Fields] |
| #4 | Toddlers [All Fields] |
| #5 | Preadolescents [All Fields] |
| #6 | Adolescent [All Fields] |
| #7 | Young [All Fields] |
| #8 | School aged [All Fields] |
| #9 | School-aged [All Fields] |
| #10 | 1 OR 2 OR 3 OR 4 OR 5 OR 6 OR7 OR 8 OR 9 |
| #11 | Diabetes Mellitus [All Fields] |
| #12 | Diabetes Mellitus, Type 1 [MeSH Terms] |
| #13 | Diabetes Mellitus, Insulin-Resistant [MeSH Terms] |
| #14 | Diabetes Mellitus, Insulin-Dependent [All Fields] |
| #15 | T1D [All Fields] |
| #16 | 11 OR 12 OR 13 OR14 OR 15 |
| #17 | Incidence [All Fields] |
| #18 | Trend [All Fields] |
| #19 | Epidemic* [All Fields] |
| #20 | 17 OR 18 OR 19 |
| #21 | observat* [All Fields] |
| #22 | cross-sectional [All Fields] |
| #23 | longitudinal [All Fields] |
| #24 | survey [All Fields] |
| #25 | 21 OR 22 OR 23 OR 24 NOT review |
| #26 | Russia [All Fields] |
| #27 | Germany [All Fields] |
| #28 | Turkey [All Fields] |
| #29 | France [All Fields] |
| #30 | United Kingdom [All Fields] |
| #31 | UK [All Fields] |
| #32 | Italy [All Fields] |
| #33 | Spain [All Fields] |
| #34 | Ukraine [All Fields] |
| #35 | Poland [All Fields] |
| #36 | Romania [All Fields] |
| #37 | Kazakhstan [All Fields] |
| #38 | Netherlands [All Fields] |
| #39 | Belgium [All Fields] |
| #40 | Greece [All Fields] |
| #41 | Czech Republic [All Fields] |
| #42 | Portugal [All Fields] |
| #43 | Sweden [All Fields] |
| #44 | Hungary [All Fields] |
| #45 | Azerbaijan [All Fields] |
| #46 | Belarus [All Fields] |
| #47 | Austria [All Fields] |
| #48 | Switzerland [All Fields] |
| #49 | Bulgaria [All Fields] |
| #50 | Serbia [All Fields] |
| #51 | Denmark [All Fields] |
| #52 | Finland [All Fields] |
| #53 | Slovakia [All Fields] |
| #54 | Norway [All Fields] |
| #55 | Ireland [All Fields] |
| #56 | Croatia [All Fields] |
| #57 | Bosnia and Herzegovina [All Fields] |
| #58 | Georgia [All Fields] |
| #59 | Moldova [All Fields] |
| #60 | Armenia [All Fields] |
| #61 | Lithuania [All Fields] |
| #62 | Albania [All Fields] |
| #63 | Macedonia [All Fields] |
| #64 | Slovenia [All Fields] |
| #65 | Latvia [All Fields] |
| #66 | Kosovo [All Fields] |
| #67 | Estonia [All Fields] |
| #68 | Cyprus [All Fields] |
| #69 | Montenegro [All Fields] |
| #70 | Luxembourg [All Fields] |
| #71 | North Macedonia [All Fields] |
| #72 | Malta [All Fields] |
| #73 | Iceland [All Fields] |
| #74 | Andorra [All Fields] |
| #75 | Liechtenstein [All Fields] |
| #76 | Monaco [All Fields] |
| #77 | San Marino [All Fields] |
| #78 | Vatican city [All Fields] |
| #79 | 26 OR 27 OR 28 OR 29 OR 30 OR 31 OR 32 OR 33 OR 34 OR 35 OR 36 OR 37 OR 38 OR 39 OR 40 OR 41 OR 42 OR 43 OR 44 OR 45 OR 46 OR 47 OR 48 OR 49 OR 50 OR 51 OR 52 OR 53 OR 54 OR 55 OR 56 OR 57 OR 58 OR 59 OR 60 OR 61 OR 62 OR 63 OR 64 OR 65 OR66 OR 67 OR 68 OR 69 OR 70 OR 71 OR 72 OR 73 OR 74 OR 75 OR 76 OR 77 OR 78 |
| #80 | 10 AND 16 AND 20 AND 25 AND 79 Filters: from 1994 – 2023 |

# Supplementary Table 2. Characteristics of studies included in the systematic review and or meta-analysis

|  | | | | | | **Population characteristics** | | **Outcome** | | | |
| --- | --- | --- | --- | --- | --- | --- | --- | --- | --- | --- | --- |
| **Reference** | **Country** | **European region** | **Level of representa-tiveness** | **Period of study** | **Study design** | **Age distribution (years)** | **Sample size** |  | **Mean annual incidence (each cases/100 000/yr)** | | |
|  |  |  |  |  |  |  |  | TOTAL | 0-4yr | 5-9yr | 10-1 4yr |
| *Rami et al 2001* | Austria | Western | Regional (Upper Austria) | 1994-1996 | Prospective | 0-30 | 137 | 8.99 (7.02-11.4) | 5.7 (3.2-4.99) | 9.9 (6.5-14.6) | 11.3 (7.6-11.3) |
| *Schober et al 2008* | Austria | Western | National | 1979-2005 | Prospective | 0-15 | 3599 | 10.3 (9.6-11.1) (1995-1999)  14.6 (13.7-15.4) (2000-2005) | 8.8 (NR) (1994-2003)  13.1 (NR) (2004-2005) | 12.5 (NR)  (1994-2003)  16.7 (NR) (2004-2005) | 13.7 (NR)  (1994-2003)  19.3 (NR)  (2004-2005) |
| *Patterson et al 2019* | Austria | Western | National | 1989-2013 | Capture-recapture method | 0-14 | 4594 | 9.8 (NR) (1994-1998)  13.2 (NR) (1999-2003)  17.3 (NR) (2004-2008)  19.7 (NR) (2009-2013) | 6.7 (NR) (boys)  5.3 (NR) (girls) (1994-1998)  12.9 (NR) (boys)  9.3 (NR) (girls) (1999-2003)  14.3 (NR) (boys) 12.6 (NR) (girls) (2004-2008)  14.3 (NR) (boys) 12.1 (NR) (girls) (2009-2013) | 10.9 (NR) (boys)  10.0 (NR) (girls) (1994-1998)  13.4 (NR) (boys)  15.2 (NR) (girls) (1999-2003)  18.0 (NR) (boys) 16.9 (NR) (girls) (2004-2008)  22.2 (NR) (boys) 19.5 (NR) (girls) (2009-2013) | 13.3 (NR) (boys)  12.7 (NR) (girls) (1994-1998)  15.3 (NR) (boys)  13.4 (NR) (girls) (1999-2003)  22.4 (NR) (boys) 19.7 (NR) (girls) (2004-2008)  26.8 (NR) (boys) 23.0 (NR) (girls) (2009-2013) |
| *Patterson et al 2019* | Belgium | Western | Regional (Antwerp) | 1989-2013 | Capture-recapture method |  | 610 | 13.0 (NR) (1994-1998)  16.4 (NR) (1999-2003)  16.3 (NR) (2004-2008)  18.1 (NR) (2009-2013) | 9.2 (NR) (boys)  8.9 (NR) (girls) (1994-1998)  12.2 (NR) (boys)  10.3 (NR) (girls) (1999-2003)  11.0 (NR) (boys) 9.3 (NR) (girls) (2004-2008)  10.3 (NR) (boys) 12.8 (NR) (girls) (2009-2013) | 13.6 (NR) (boys)  11.9 (NR) (girls) (1994-1998)  22.3 (NR) (boys)  15.7 (NR) (girls) (1999-2003)  15.8 (NR) (boys) 16.4 (NR) (girls) (2004-2008)  22.2 (NR) (boys) 20.2 (NR) (girls) (2009-2013) | 18.0 (NR) (boys)  16.6 (NR) (girls) (1994-1998)  20.8 (NR) (boys)  17.1 (NR) (girls) (1999-2003)  24.7 (NR) (boys) 20.6 (NR) (girls) (2004-2008)  22.5 (NR) (boys) 20.5 (NR) (girls) (2009-2013) |
| *Bratina et al 2001* | Bosnia and Herzegovina | Eastern | Regional (Tuzla) | 1990-1998 | Prospective | 0-14 | 43 | 3.03 (2.0-4.1) | 0.80 (0-1.7) | 4.68 (2.5-6.8) | 5.16 (2.8-7.5) |
| *Bukara-Radujkovic et al 2018* | Bosnia and Herzegovina | Eastern | Regional (Republica Sprska) | 2001-2016 | Retrospective based on the capture-recapture method | 0-14 | 294 | 11.0 (9.8-12.4) | 5.8 (NR) | 13.5 (NR) | 13.6 (NR) |
| *Stipancic et al 2008* | Croatia | Eastern | National | 1995-2003 | Retrospective (from 1995 to 1998) and Prospective (from 1999 to 2003) | 0-14 | 692 | 9.05 (8.38-9.72) | 5.77 (4.79-6.74) | 9.80 (8.60-11.01) | 11.13 (9.88-12.38) |
| *Rojnic et al 2014* | Croatia | Eastern | National | 2004-2012 | Prospective | 0-14 | 1066 | 17.44 (16.39-18.49) | 13.09 (11.46-14.72) | 18.66 (16.76-20.55) | 20.04 (18.18-21.90) |
| *Patterson et al 2019* | Croatia | Eastern | Regional (Zagreb) | 1989-2013 | Capture-recapture method | 0-14 | 364 | 11.1 (NR) (1994-1998)  14.8 (NR) (1999-2003)  19.4 (NR) (2004-2008)  18.8 (NR) (2009-2013) | 7.8 (NR) (boys)  6.8 (NR) (girls) (1994-1998)  14.2 (NR) (boys)  12.2 (NR) (girls) (1999-2003)  13.6 (NR) (boys) 12.4 (NR) (girls) (2004-2008)  16.0 (NR) (boys) 13.6 (NR) (girls) (2009-2013) | 11.3 (NR) (boys)  12.7 (NR) (girls) (1994-1998)  17.6 (NR) (boys)  20.4 (NR) (girls) (1999-2003)  22.0 (NR) (boys) 22.5 (NR) (girls) (2004-2008)  24.2 (NR) (boys) 24.1 (NR) (girls) (2009-2013) | 15.4 (NR) (boys)  15.1 (NR) (girls) (1994-1998)  19.7 (NR) (boys)  18.2 (NR) (girls) (1999-2003)  25.5 (NR) (boys) 20.1 (NR) (girls) (2004-2008)  27.8 (NR) (boys) 25.1 (NR) (girls) (2009-2013) |
| *Mousa et al 2020* | Cyprus | Southern | National | 2001-2016 | Retrospective | 0-16 | 107 | 11.1 (NR) | 5.75 (NR) | 15.4 (NR) (5-8 yrs) | 17.6 (NR) (9-12 yrs) |
| *Toumba et al 2007* | Cyprus | Southern | National | 1990-2004 | Prospective and capture-recapture method | 0-14 | 277 | 10.48 (8.4-13.0) (1995-1999)  14.87 (12.2-18.0) (2000-2004) | NR | NR | NR |
| *Cinek et al 2000* | Czech Republic | Eastern | National | 1990-1997 | Prospective | 0-14 | 1604 | 10.1 (9.6-10.6) | 5.9 (5.3-6.7) | 10.5 (9.7-11.5) | 13.1 (12.2-14.1) |
| *Cinek et al 2012* | Czech Republic | Eastern | National | 1989-2009 | Prospective | 0-14 | 5155 | 14.4 (NR) (<2004)  19.2 (NR) | 10.3 (NR) (<2004)  12.3 (NR) | 15.5 (NR) (<2004)  22.0 (NR) | 17.2 (NR) (<2004)  22.9 (NR) |
| *Cinek et al 2022* | Csech Republic | Eastern | National | 2010-2019 | Prospective | <15 | NR | 27.2(NR) | NR | NR | NR |
| *Patterson et al 2019* | Denmark | Northern | National | 1989-2013 | Capture-recapture method | 0-14 | 4148 | 16.3 (NR) (1994-1998)  22.5 (NR) (1999-2003)  25.2 (NR) (2004-2008)  27.0 (NR) (2009-2013) | 10.6 (NR) (boys)  12.2 (NR) (girls) (1994-1998)  11.5 (NR) (boys)  14.1 (NR) (girls) (1999-2003)  14.4 (NR) (boys) 14.1 (NR) (girls) (2004-2008)  16.2 (NR) (boys) 12.6 (NR) (girls) (2009-2013) | 14.1 (NR) (boys)  17.4 (NR) (girls) (1994-1998)  22.9 (NR) (boys)  23.9 (NR) (girls) (1999-2003)  23.2 (NR) (boys) 26.8 (NR) (girls) (2004-2008)  28.5 (NR) (boys) 30.5 (NR) (girls) (2009-2013) | 18.8 (NR) (boys)  24.8 (NR) (girls) (1994-1998)  34.4 (NR) (boys)  24.8 (NR) (girls) (1999-2003)  38.8 (NR) (boys) 34.0 (NR) (girls) (2004-2008)  37.2 (NR) (boys) 36.8 (NR) (girls) (2009-2013) |
| *Teeäär et al 2010* | Estonia | Northern | National | 1999-2006 | Prospective-retrospective | 0-15 | 310 | 17.2 (13.1-21.2) | 15.7 (12.6-19.5) | 21.2 (17.7-25.3) | 14.5 (12.0-17.4) |
| *Podar et al 2001* | Estonia | Northern | National | 1983-1998 | Prospective | 0-14 | 301 | 11.2 (10.3-12.1) | 10.3 (7.2-14.2) (1991-1998; boys)  7.5 (4.9-11.1) (1991-1998; girls) | 11.7 (8.7-15.3) (1991-1998; boys)  9.6 (6.9-13.0) (1991-1998; girls) | 16.5 (12.9-20.7) (1991-1998; boys)  16.8 (13.2-21.1) (1991-1998; girls) |
| *Knip,et al 2023* | Finland | Northern | National | 2020-2021 | Prospective | <15 | 785 | 61.0 (56.8-65.4) during pandemic | 47.0 (40.2–54.5) | 70.0 (62.4–78.2) | 63.5 (56.5–71.1) |
| *Rytkönen et al 2003* | Finland | Northern | National | 1987-1996 | Prospective | 0-14 | 3607 | 37.4 (36.0-38.5)  (1987-1996)  35.9 (34.1-37.5) (1987-1991)  38.8 (37.0-40.5) (1992-1996) | NR | NR | NR |
| *Parviainen et al 2020* | Finland | Northern | National | 2003-2018 | Prospective | 0-14 | 7871 | 57.9 (55.4-60.4) (2003-2006)  56.0 (53.6-59.6) (2007-2010)  53.4 (51.1-55.9) (2011-2014)  52.2 (49.8-54.6) (2015-2018) | 51.1 (47.1-55.5) (2003-2006)  50.4 (46.5-54.6) (2007-2010)  43.1 (39.4-46.9) (2011-2014)  39.3 (35.7-43.1) (2015-2018) | 68.2 (63.6-73.1) (2003-2006)  62.3 (57.9-67.1) (2007-2010)  59.4 (55.1-63.9) (2011-2014)  58.6 (54.4-63.0) (2015-2018) | 54.3 (50.4-58.5) (2003-2006)  55.6 (51.5-59.9) (2007-2010)  58.1 (53.8-62.7) (2011-2014)  57.6 (53.4-62.1) (2015-2018) |
| *Podar et al 2001* | Finland | Northern | National | 1983-1998 | Prospective | 0-14 | 3151 | 37.7 (36.7-38.7) | 31.3 (28.3-34.4) (1991-1998; boys)  32.4 (29.3-35.7) (1991-1998; girls) | 45.1 (41.5-48.9) (1991-1998; boys)  47.1 (43.4-51.1) (1991-1998; girls) | 49.2 (45.5-53.1) (1991-1998; boys)  39.3 (35.9-42.9) (1991-1998; girls) |
| *Mauny et al 2005* | France | Western | Regional (Franche Comté) | 1980-1998 | Prospective- retrospective based on a capture-recapture method | 0-14 | 308 | 7.09 (6.32-7.93) | 6.03 (4.76-7.53) | 6.45 (5.23-7.88) | 8.56 (7.18-10.13) |
| *Barat et al 2008* | France | Western | Regional (Aquitaine) | 1988-2004 | Prospective | 0-14 | 897 | 10.5 (9.81-11.19) | NR | NR | NR |
| *Piffaretti et al 2019* | France | Western | National | 2010-2015 | Prospective | 6 months-14 yr | 12067 | 15.4 (14.7-16.1) (2010)  19.1 (18.3-19.9) (2015) | 14.2 (12.9-15.4) (2015; 6 months-4 yr; boys)  13.8 (12.1-15.5) (2015; 6 months-4 yr; girls) | 19.4 (18.1-20.8) (2015; boys)  18.7 (16.8-20.5) (2015; girls) | 23.1(21.7-24.6) (2015; boys)  22.8 (20.8-24.9) (2015; girls) |
| *Baechle et al 2023* | Germany | Western | National | 2020-2021 | Prospective | <18 | 30840 | 19.8 (19.5–20.0) 2011-2019 period  24.9 (24.0–25.7)  2020 year  27.0 (26.1–27.9)  2021 | NR | NR | NR |
| *Bendas et al 2015* | Germany | Western | National and 3 regional (North-Rhine-Westphalia, Baden-Wuerttembert, Saxony) | 1999-2008 | Prospective-retrospective | 0-14 | - | 19.4 (18.7-20.1) (1999-2003)  22.9 (22.1-23.7) (2004-2008) | 14.5 (14.0-15.1) (1999-2003)  17.1 (16.5-17.8) (2004-2008) | 21.5 (20.1-22.9) (1999-2003)  25.4 (23.8-27.1) (2004-2008) | 22.2 (20.8-23.7) (1999-2003)  26.3 (24.6-28.0) (2004-2008) |
| *Ehehalt et al 2008* | Germany | Western | Regional (Baden-Württemberg) | 1987-2003 | Prospective-retrospective | 0-14 | 4017 | 14.1 (13.7-14.6) | NR | NR | NR |
| *Galler et al 2010* | Germany | Western | Regional (Saxony) | 1999-2008 | Prospective and capture-recapture method | 0-14 | 827 | 17.5 (16.3-18.7)^2^ | 12.9 (11.2-14.7) ^2^ | 18.7 (16.5-21.0)^2^ | 20.7 (18.6-22.9) ^2^ |
| *Rosenabuer et al 2002* | Germany | Western | National and Regional (Former Eastern Germany, Baden-Württemberg, Düsseldorf region, Germany) | 1993-1995 | Prospective-retrospective | 0-19 | 2615 | 14.2 (12.9-15.5) | 8.1 (7.6-8.6) | 15.3 (13.1-17.9) | 18.5 (15.9-21.5) |
| *Patterson et al 2019* | Germany | Western | Regional (Baden W) | 1989-2013 | Capture-recapture method | 0-14 | 6931 | 13.0 (NR) (1994-1998)  15.4 (NR) (1999-2003)  22.0 (NR) (2004-2008)  23.1 (NR) (2009-2013) | 9.6 (NR) (boys)  8.6 (NR) (girls) (1994-1998)  11.9 (NR) (boys)  11.1 (NR) (girls) (1999-2003)  18.7 (NR) (boys) 16.8 (NR) (girls) (2004-2008)  18.0 (NR) (boys) 16.7 (NR) (girls) (2009-2013) | 12.7 (NR) (boys)  13.5 (NR) (girls) (1994-1998)  16.0 (NR) (boys)  17.8 (NR) (girls) (1999-2003)  22.8 (NR) (boys) 25.1 (NR) (girls) (2004-2008)  26.1 (NR) (boys) 25.0 (NR) (girls) (2009-2013) | 16.4 (NR) (boys)  17.1 (NR) (girls) (1994-1998)  19.3 (NR) (boys)  16.3 (NR) (girls) (1999-2003)  27.3 (NR) (boys) 21.1 (NR) (girls) (2004-2008)  31.8 (NR) (boys) 21.2 (NR) (girls) (2009-2013) |
| *Weis et al 2023* | Germany | Western | Regional (Bavaria) | 2018-2021 | Prospective | 0-14 | 1242 | 29.9 (27.7-32.2) between January 2020 and December 2021 19.5 (17.8-21.4) between January 2018 and December 2019 | NR | NR | NR |
| *Mamoulakis et al 2018* | Greece | Southern | Regional (Crete) | 1992-2016 | Prospective | 0-14 | 271 | 10.5 ( 9.2-11.8) | 7.0 (5.3-9.0) | 12.3 (10.1-14.9) | 11.9 (9.9-14.4) |
| *Gyurus et al 2012* | Hungary | Eastern | National (excluded Budapest and the surrounding region) | 1989-2009 | Prospective and capture-recapture method | 0-14 | 3432 | 12.5 (12.0-12.9) | 8.8 (8.2-9.5) | 13.5 (12.7-14.3) | 15.1 (14.3-15.8) |
| *Patterson et al 2019* | Hungary | Eastern | National – except Budapest | 1989-2013 | Capture-recapture method | 0-14 | 4283 | 10.7 (NR) (1994-1998)  12.4 (NR) (1999-2003)  18.3 (NR) (2004-2008)  20.0 (NR) (2009-2013) | 8.0 (NR) (boys)  7.2 (NR) (girls) (1994-1998)  8.3 (NR) (boys)  9.5 (NR) (girls) (1999-2003)  14.0 (NR) (boys) 14.0 (NR) (girls) (2004-2008)  15.0 (NR) (boys) 14.0 (NR) (girls) (2009-2013) | 10.4 (NR) (boys)  12.1 (NR) (girls) (1994-1998)  12.7 (NR) (boys)  13.8 (NR) (girls) (1999-2003)  21.1 (NR) (boys) 20.6 (NR) (girls) (2004-2008)  22.5 (NR) (boys) 21.2 (NR) (girls) (2009-2013) | 12.4 (NR) (boys)  13.9 (NR) (girls) (1994-1998)  16.6 (NR) (boys)  13.5 (NR) (girls) (1999-2003)  22.3 (NR) (boys) 17.8 (NR) (girls) (2004-2008)  26.0 (NR) (boys) 21.5 (NR) (girls) (2009-2013) |
| *Bruno et al 2010* | Italy | Southern | National | 1990-2003 | Prospective and capture-recapture method | 0-14 | 5180 | 12.26 (11.93-12.60) | 9.22 (NR) | 14.0 (NR) | 15.15 (NR) |
| *Fortunato et al 2016* | Italy | Southern | Regional: (Apulia) | 2009-2013 | Retrospective | < 18 | 917 | 25.2 (NR) | 9.6 (5.0-14.1) (< 1 yr)  20.1 (16.8-23.3) (1-4 yrs) | 29.7 (26.3-33.1) (5-9 yrs) | 28.2 (25.0-31.4) (10-14 yrs) |
| *Giorda et al 2023* | Italy | Southern | Regional (Piedmont) | 2017-2021 | Retrospective | 0-29 | 834 | 13.69 (NR)  2017-2019 year  16.31 (NR)  2020-2021 year | NR | NR | NR |
| *Gesuita el al 2023* | Italy | Southern | Regional Central and Northern Italy (Marche and Piedmont) | 1989-2019 | Capture-recapture method | 6 months-14 years | 4009 | 16.9 (NR) | 12.2 (11.4-13.0) | 17.7 (16.8-18.6) | 19.9 (19.0-20.9) |
| *Passanisi et al 2022* | Italy | Southern | Regional (Calabria) | 2019-2021 | Retrospective | <15 | 163 | 20.6 17.6 – 24.1) | 15.7 (11.1– 21.6) | 24.1 (18.6 -30.8) | 21.6 (16.5 – 27.7) |
| *Patterson et al 2019* | Italy | Southern | Regional (Marche) | 1989-2013 | Capture-recapture method | 0-14 | 579 | 11.9 (NR) (1994-1998)  12.6 (NR) (1999-2003)  12.6 (NR) (2004-2008)  11.6 (NR) (2009-2013) | 10.5 (NR) (boys)  12.5 (NR) (girls) (1994-1998)  8.8 (NR) (boys)  8.1 (NR) (girls) (1999-2003)  9.9 (NR) (boys) 11.8 (NR) (girls) (2004-2008)  10.5 (NR) (boys) 7.6 (NR) (girls) (2009-2013) | 10.8 (NR) (boys)  10.1 (NR) (girls) (1994-1998)  13.0 (NR) (boys)  19.0 (NR) (girls) (1999-2003)  11.1 (NR) (boys) 15.1 (NR) (girls) (2004-2008)  8.5 (NR) (boys) 18.7 (NR) (girls) (2009-2013) | 15.1 (NR) (boys)  12.2 (NR) (girls) (1994-1998)  16.2 (NR) (boys)  10.7 (NR) (girls) (1999-2003)  15.0 (NR) (boys) 12.9 (NR) (girls) (2004-2008)  12.4 (NR) (boys) 12.1 (NR) (girls) (2009-2013) |
| *Roche et al 2002* | Ireland | Western | National | 1997 | Prospective and capture-recapture method | 0-14 | 140 | 16.3 (12.6-20.7) | 10.8 (7.1-15.7) (0-4.99 yrs) | 21.3 (16.2-27.4) (5- 9.99 yrs) | 17.0 (12.8-22.2) (10-14.99 yrs) |
| *Roche et al 2016* | Ireland | Western | National | 2008-2013 | Prospective and capture-recapture method | 0-14 | 1566 | 25.2 (22.06-28.34) (2008)  28.4 (25.11-31.69) (2013) | 20.2 (14.2-28.6) (2008; boys)  17.1 (11.3-24.8) (2008; girls)  20.9 (14.9-28.6) (2013; boys)  22.9 (16.4-31.0) (2013; girls) | 28.8 (21.0-38.5) (2008; boys)  28.0 (20.2-37.9) (2008; girls)  28.9 (21.3-38.1) (2013; boys)  29.4 (21.7-39.0) (2013; girls) | 38.5 (29.1-50.1) (2008; boys)  31.9 (23.2-42.8) (2008; girls)  38.0 (29.0-49.0) (2013; boys)  32.4 (24.0-42.9) (2013; girls) |
| *Patterson et al 2019* | Ireland | Western | National | 1989-2013 | Capture-recapture method | 0-14 | 1566 | 27.5 (NR) (2004-2008)  27.5 (NR) (2009-2013) | 20.5 (NR) (boys) 17.1 (NR) (girls) (2004-2008)  17.7 (NR) (boys) 17.3 (NR) (girls) (2009-2013) | 28.8 (NR) (boys) 28.0 (NR) (girls) (2004-2008)  30.2 (NR) (boys) 30.7 (NR) (girls) (2009-2013) | 38.5 (NR) (boys) 31.9 (NR) (girls) (2004-2008)  35.6 (NR) (boys) 33.6 (NR) (girls) (2009-2013) |
| *Podar et al 2001* | Latvia | Eastern | National | 1983-1998 | Prospective | 0-14 | 310 | 6.9 (6.4-7.5) | 4.3 (2.9-6.3) (1991-1998; boys)  4.3 (2.8-6.3) (1991-1998; girls) | 6.7 (5.0-8.9) (1991-1998; boys)  8.7 (6.7-11.1) (1991-1998; girls) | 9.8 (7.7-12.4) (1991-1998; boys)  9.3 (7.2-11.9) (1991-1998; girls) |
| *Podar et al 2001* | Lithuania | Eastern | National | 1983-1998 | Prospective | 0-14 | 505 | 7.3 (6.9-7.8) | 4.2 (3.0-5.6) (1991-1998;boys)  3.6 (2.5-5.1) (1991-1998; girls) | 5.7 (4.4-7.2) (1991-1998; boys)  9.5 (7.8-11.5) (1991-1998; girls) | 11.9 (9.9-14.1) (1991-1998; boys)  11.2 (9.3-13.4) (1991-1998; girls) |
| *Urbonaite et al 2002* | Lithuania | Eastern | National | 1983-1998 | Prospective | 0-14 | 972 | 7.36 (6.90-7.82)^2^ | 4.0 (2.0-6.0) (boys)  3.5 (1.75-3.8) (girls) | 7.0 (5.5-9.0) (boys)  9.0 (7.0-11.0) (girls) | 10.5 (8.75-12.25) (boys)  10.0 (8.0-12.0) (girls) |
| *Patterson et al 2019* | Lithuania | Eastern | National | 1989-2013 | Capture-recapture method | 0-14 | 1862 | 8.2 (NR) (1994-1998)  10.3 (NR) (1999-2003)  14.2 (NR) (2004-2008)  19.9 (NR) (2009-2013) | 4.3 (NR) (boys)  4.8 (NR) (girls) (1994-1998)  8.7 (NR) (boys)  7.3 (NR) (girls) (1999-2003)  9.4 (NR) (boys) 9.1 (NR) (girls) (2004-2008)  14.1 (NR) (boys) 12.0 (NR) (girls) (2009-2013) | 5.0 (NR) (boys)  10.3 (NR) (girls) (1994-1998)  8.4 (NR) (boys)  11.6 (NR) (girls) (1999-2003)  12.6 (NR) (boys) 17.9 (NR) (girls) (2004-2008)  15.0 (NR) (boys) 26.9 (NR) (girls) (2009-2013) | 12.8 (NR) (boys)  12.1 (NR) (girls) (1994-1998)  13.6 (NR) (boys)  12.5 (NR) (girls) (1999-2003)  18.9 (NR) (boys) 17.5 (NR) (girls) (2004-2008)  29.1 (NR) (boys) 22.3 (NR) (girls) (2009-2013) |
| *Patterson et al 2019* | Luxembourg | Western | National | 1989-2013 | Capture-recapture method | 0-14 | 312 | 12.3 (NR) (1994-1998)  15.7 (NR) (1999-2003)  18.4 (NR) (2004-2008)  18.6 (NR) (2009-2013) | 7.1 (NR) (boys)  7.5 (NR) (girls) (1994-1998)  5.5 (NR) (boys)  10.2 (NR) (girls) (1999-2003)  8.3 (NR) (boys) 17.8 (NR) (girls) (2004-2008)  12.0 (NR) (boys) 8.4 (NR) (girls) (2009-2013) | 15.5 (NR) (boys)  14.6 (NR) (girls) (1994-1998)  11.0 (NR) (boys)  21.7 (NR) (girls) (1999-2003)  16.0 (NR) (boys) 8.4 (NR) (girls) (2004-2008)  23.5 (NR) (boys) 22.3 (NR) (girls) (2009-2013) | 13.5 (NR) (boys)  16.0 (NR) (girls) (1994-1998)  27.0 (NR) (boys)  18.8 (NR) (girls) (1999-2003)  32.0 (NR) (boys) 28.0 (NR) (girls) (2004-2008)  25.3 (NR) (boys) 20.0 (NR) (girls) (2009-2013) |
| *Formosa et al 2012* | Malta | Southern | National | 2006-2010 | Prospective | 0-14 | 81 | 24.68 (21.94-27.43) | 21.7 (8.9-34.5) | 30.4 (9.8-51.0) | 16.1 (10.9-21.4) |
| *Raicevic et al 2022* | Montenegro | Eastern | National | 1991-2020 | Retrospective | <15 | 582 | 15.6 (1.44–4.69) | 11.1 (0.93–1.31) | 17.5 (1.52–2.00) | 18.0 (1.58–2.05) |
| *Samardzic et al 2011* | Montenegro | Eastern | National | 1997-2011 | Prospective and capture-recapture method | 0-14 | - | 10.6 (7.2-14.0) (1997-2001)  15.8 (11.7-19.8) (2002-2006)  18.6 (13.0-24.1) (2007-2011) | 8.1 (4.9-11.2) (1997-2001)  9.9 (3.1-6.6) (2002-2006)  14.0 (3.7-24.4) (2007-2011) | 10.3 (6.3-14.2) (1997-2001)  18.3 (14.3-22.2) (2002-2006)  24.0 (13.1-34.9) (2007-2011) | 14.0 (6.9-21.1) (1997-2001)  20.6 (15.0-26.2) (2002-2006)  19.0 (12.4-25.6) (2007-2011) |
| *Patterson et al 2019* | Montenegro | Eastern | National | 1989-2013 | Capture-recapture method | 0-14 | 362 | 10.1 (NR) (1994-1998)  14.7 (NR) (1999-2003)  17.4 (NR) (2004-2008)  18.5 (NR) (2009-2013) | 5.7 (NR) (boys)  9.3 (NR) (girls) (1994-1998)  13.3 (NR) (boys)  11.2 (NR) (girls) (1999-2003)  10.9 (NR) (boys) 10.7 (NR) (girls) (2004-2008)  18.8 (NR) (boys) 10.9 (NR) (girls) (2009-2013) | 9.3 (NR) (boys)  8.5 (NR) (girls) (1994-1998)  10.7 (NR) (boys)  21.0 (NR) (girls) (1999-2003)  18.7 (NR) (boys) 20.9 (NR) (girls) (2004-2008)  21.9 (NR) (boys) 29.1 (NR) (girls) (2009-2013) | 17.4 (NR) (boys)  10.5 (NR) (girls) (1994-1998)  16.7 (NR) (boys)  15.1 (NR) (girls) (1999-2003)  25.9 (NR) (boys) 17.6 (NR) (girls) (2004-2008)  18.5 (NR) (boys) 11.9 (NR) (girls) (2009-2013) |
| *Spaans et al 2015* | Netherlands | Western | National | 2010-2011 | Retrospective | 0-14 | 1243 | 21.4 (20.2-22.6) | 12.4 (12.0-13.9) | 23.6 (21.5-25.8) | 27.6 (25.3-30.0) |
| *Fazeli Farsani et al 2016* | Netherlands | Western | National | 1999-2011 | Prospective | 0-19 | 1213 | 18.1 (16.6-19.6) (1999)  24.9 (23.1-26.7) (2011) | NR | NR | NR |
| *Patterson et al 2019* | North Macedonia | Eastern | National | 1989-2013 | Capture-recapture method | 0-14 | 584 | 3.9 (NR) (1994-1998)  6.0 (NR) (1999-2003)  7.2 (NR) (2004-2008)  7.7 (NR) (2009-2013) | 1.8 (NR) (boys)  0.8 (NR) (girls) (1994-1998)  3.3 (NR) (boys)  2.9 (NR) (girls) (1999-2003)  7.1 (NR) (boys) 6.1 (NR) (girls) (2004-2008)  3.7 (NR) (boys) 7.6 (NR) (girls) (2009-2013) | 5.1 (NR) (boys)  6.0 (NR) (girls) (1994-1998)  5.8 (NR) (boys)  7.3 (NR) (girls) (1999-2003)  8.9 (NR) (boys) 8.2 (NR) (girls) (2004-2008)  7.8 (NR) (boys) 10.1 (NR) (girls) (2009-2013) | 6.1 (NR) (boys)  3.5 (NR) (girls) (1994-1998)  8.0 (NR) (boys)  8.8 (NR) (girls) (1999-2003)  6.9 (NR) (boys) 5.9 (NR) (girls) (2004-2008)  8.9 (NR) (boys) 8.2 (NR) (girls) (2009-2013) |
| *Aamodt et al 2007* | Norway | Northern | National | 1973-1982 and 1989-2003 | Retrospective and prospective | 0-19 | 5035 | 22.7 (22.1-23.4) (overall 0-19 yrs) | 10.1 (9.1-11.0) (0-2 yrs)  19.0 (17.8-20.3) (3-5 yrs) | 25.1 (23.7-26.6) (6-8 yrs) | 31.1 (29.5-32.8) (9-11 yrs)  28.8 (26.5-29.6) (12-14 years) |
| *Skrivarhaug et al 2014* | Norway | Northern | National | 1989-2012 | Prospective and capture-recapture method | 0-14 | 2686 (2004-2012) | 22.6 (21.4-23.7) (1989-1996)  28.4 (27.3-29.6) (1996-2004)  32.7 (31.5-34.0) (2004-2012) | 19.2 (17.6-21.0) (2004-2012) | 35.2 (33.0-37.5) (2004-2012) | 43.1 (40.7-45.5) (2004-2012) |
| *Patterson et al 2019* | Norway | Northern | National | 1989-2013 | Capture-recapture method | 0-14 | 6348 | 24.6 (NR) (1994-1998)  29.6 (NR) (1999-2003)  33.1 (NR) (2004-2008)  33.6 (NR) (2009-2013) | 14.9 (NR) (boys)  13.2 (NR) (girls) (1994-1998)  18.5 (NR) (boys)  19.5 (NR) (girls) (1999-2003)  21.0 (NR) (boys) 17.8 (NR) (girls) (2004-2008)  21.5 (NR) (boys) 18.6 (NR) (girls) (2009-2013) | 30.7 (NR) (boys)  27.7 (NR) (girls) (1994-1998)  32.0 (NR) (boys)  32.9 (NR) (girls) (1999-2003)  34.4 (NR) (boys) 39.2 (NR) (girls) (2004-2008)  34.7 (NR) (boys) 37.4 (NR) (girls) (2009-2013) | 33.6 (NR) (boys)  27.7 (NR) (girls) (1994-1998)  41.9 (NR) (boys)  32.7 (NR) (girls) (1999-2003)  46.7 (NR) (boys) 39.8 (NR) (girls) (2004-2008)  49.2 (NR) (boys) 40.1 (NR) (girls) (2009-2013) |
| *Jarosz-Chobot et al 2011* | Poland | Eastern | Regional (Łódzkie, Małopolski, Podlasie, Pomorskie, Silesia, Warmia-Mazury and Podkarpackie) | 1989-2004 | Prospective | 0-14 | 4268 | 10.2 (8.7-11.87) ^2^ | 6.2 (5.0-7.5) ^2^ | 10.9 (9.4-12.6) ^2^ | 12.6 (10.9-14.4) ^2^ |
| *Szalecki et al 2016* | Poland | Eastern | Regional (Eastern and central Poland) | 2010-2014 | Prospective and retrospective (cohort study) | < 18 | 2174 | 14.72 (13.08-16.36) (2010) 21.27 (19.27-23.26) (2014) | 11.48 (9.01-13.95) ( 2010)  15.32 (12.41-18.24) (2014) | 16.02 (12.94-19.09) (2010)  21.18 (17.78-24.59) (2014) | 16.80 (13.82-19.78) (2010)  27.63 (23.61-31.66) (2014) |
| *Chobot et al 2017* | Poland | Eastern | Regional | 1989-2004 and 2005-2012 | Prospective | < 15 | 7248 | 20.22 (17.23-23.59) ^2^ (2005-2012) | 14.93 (12.38-17.87) ^2^ (2005-2012) | 23.02 (19.82-26.60) ^2^ (2005-2012) | 21.81 (18.70-25.30) ^2^ (2005-2012) |
| *Patterson et al 2019* | Poland | Eastern | Regional (Katowice) | 1989-2013 | Capture-recapture method | 0-14 | 2360 | 7.9 (NR) (1994-1998)  13.0 (NR) (1999-2003)  16.5 (NR) (2004-2008)  20.1 (NR) (2009-2013) | 3.8 (NR) (boys)  3.7 (NR) (girls) (1994-1998)  8.8 (NR) (boys)  7.5 (NR) (girls) (1999-2003)  11.7 (NR) (boys) 12.3 (NR) (girls) (2004-2008)  13.4 (NR) (boys) 14.8 (NR) (girls) (2009-2013) | 8.0 (NR) (boys)  8.7 (NR) (girls) (1994-1998)  13.0 (NR) (boys)  15.4 (NR) (girls) (1999-2003)  19.6 (NR) (boys) 18.5 (NR) (girls) (2004-2008)  19.0 (NR) (boys) 25.3 (NR) (girls) (2009-2013) | 9.9 (NR)(boys)  13.2 (NR) (girls) (1994-1998)  19.2 (NR) (boys)  14.1 (NR) (girls) (1999-2003)  18.3 (NR) (boys) 18.5 (NR) (girls) (2004-2008)  27.4 (NR) (boys) 20.6 (NR) (girls) (2009-2013) |
| *Serban et al 2015* | Romania | Eastern | National | 2002-2011 | Retrospective | < 18 | 3196 | 8.0 (7.6-8.4)^3^ (0-14 yrs) | 4.8 (4.4-5.3) ^3^ | 8.8 (8.2-9.4) ^3^ | 9.6 (9.0-10.1) |
| *Vlad et al 2018* | Romania | Eastern | National | 1996-2015 | Retrospective | 0-14 | 5422 | 7.2 (7.0-7.4) ^3^ | 4.5 (4.2-4.7) ^3^ | 7.6 (7.3-8.0) ^3^ | 9.1 (8.7-9.4) ^3^ |
| *Patterson et al 2019* | Romania | Eastern | Bucharest | 1989-2013 | Capture-recapture method | 0-14 | 714 | 5.6 (NR) (1994-1998)  9.7 (NR) (1999-2003)  12.3 (NR) (2004-2008)  13.4 (NR) (2009-2013) | 2.2 (NR)(boys)  3.2 (NR) (girls) (1994-1998)  6.3 (NR) (boys)  4.1 (NR) (girls) (1999-2003)  8.4 (NR) (boys) 5.0 (NR) (girls) (2004-2008)  11.4 (NR) (boys) 10.2 (NR) (girls) (2009-2013) | 8.0 (NR) (boys)  7.1 (NR) (girls) (1994-1998)  9.6 (NR) (boys)  14.8 (NR) (girls) (1999-2003)  14.5 (NR) (boys) 17.5 (NR) (girls) (2004-2008)  13.3 (NR) (boys) 12.9 (NR) (girls) (2009-2013) | 6.6 (NR) (boys)  6.6 (NR) (girls) (1994-1998)  9.9 (NR) (boys)  13.4 (NR) (girls) (1999-2003)  13.2 (NR) (boys) 15.3 (NR) (girls) (2004-2008)  15.9 (NR) (boys) 16.8 (NR) (girls) (2009-2013) |
| *Vorgučin* *et al 2022* | Serbia | Eastern | Regional (Vojvodina) | 2017-2021 | Retrospective | <19 | 231 | 10.59 () 2017 year  17.30() 2021 year | NR | NR | NR |
| *Sipetic et al 2013* | Serbia | Eastern | Regional (Belgrade) | 1982-2005 | Retrospective | 0-14 | 702 | 10.4 (9.6-11.2) | 5.5 (4.5-6.7) | 11.9 (10.5-13.5) | 15.4 (13.8-17.1) |
| *Bratina et al 2001* | Slovenia | Eastern | National | 1990-1998 | Prospective | 0-14 | 229 | 8.54 (7.5-9.5) | 6.17 (4.6-7.7) | 9.20 (7.4-10.9) | 10.79 (9.0-12.6) |
| *Patterson et al 2019* | Slovenia | Eastern | National | 1989-2013 | Capture-recapture method | 0-14 | 949 | 9.1 (NR) (1994-1998)  11.1 (NR) (1999-2003)  14.9 (NR) (2004-2008)  16.3 (NR) (2009-2013) | 7.9 (NR)(boys)  5.0 (NR) (girls) (1994-1998)  4.7 (NR) (boys)  9.1 (NR) (girls) (1999-2003)  9.4 (NR) (boys) 14.9 (NR) (girls) (2004-2008)  10.2 (NR) (boys) 13.5 (NR) (girls) (2009-2013) | 10.5 (NR)(boys)  11.4 (NR) (girls) (1994-1998)  11.3 (NR) (boys)  12.7 (NR) (girls) (1999-2003)  14.8 (NR) (boys) 18.0 (NR) (girls) (2004-2008)  18.1 (NR) (boys) 18.2 (NR) (girls) (2009-2013) | 8.9 (NR)(boys)  10.9 (NR) (girls) (1994-1998)  15.1 (NR) (boys)  13.8 (NR) (girls) (1999-2003)  14.7 (NR) (boys) 17.6 (NR) (girls) (2004-2008)  16.7 (NR) (boys) 20.9 (NR) (girls) (2009-2013) |
| *Bahillo et al 2007* | Spain | Southern | Regional (Castilla-Leon) | 2003-2004 | Prospective | 0-14 | 130 | 22.2 (14.6-29.8) | 14.7 (9.1-20.4) | 32.5 (24.3-40.6) | 19.3 (13.5-25.1) |
| *Fernández-Ramos et al*. *2017* | Spain | Southern | Regional (Biscay) | 1990-2013 | Prospective-retrospective based on the capture-recapture method | 0-14 | 399 | 10.7 (9.6-11.7) | 5.1 (3.8-6.5) | 14.6 (12.4-16.8) | 13.2 (11.3-15.2) |
| *Forga-Llenas et al 2018* | Spain | Southern | Regional (Navarra) | 1977-2016 | Prospective-retrospective | 0-14 | 577 | 14.99 (13.79-16.26) | 9.33 (7.69-11.22) | 13.95 (11.98-16.15) | 21.03 (18.66-23.61) |
| *Gimeno-Benítez et al 2014* | Spain | Southern | Regional (Extremadura) | 2003-2007 | Based on the capture-recapture method | 0-14 | 208 | 25.5 (22.1-29.0) | 18.5 (10.1-30.3) | 25.2 (20.1-29.4) | 31.8 (25.8-34.1) |
| *Giralt-Muiña et al 2012* | Spain | Southern | Regional (Castilla-La Mancha) | 2007-2008 | Prospective based on the capture-recapture method | 0-14 | 81 | 27.6 (21.59-33.61) | NR | NR | NR |
| *Goñi-Iriarte et al. 2023* | Spain | Southern | Regional (Navarra) | 2009-2020 | Prospective | <15 | 627 | 20.6 (18.2-23.3) | 13.4 (10.1-17.6) | 20.6 (16.5) | 27.8(22.8-33.5) |
| *Hernández-Herrero et al 2021* | Spain | Southern | Regional (Tarragona) | 2018-2020 | Retrospective | <15 | 99 | 29.2cases per 100,000 inhabitants in 2020, 18.1/100,000in 2019 and 22.9/100,000 in 2018 (95% CI: 17.1---29.7) | NR | NR | NR |
| *López-Siguero et al 2002* | Spain | Southern | Regional (Malaga) | 1982-2000 | Capture-recapture method | 0-14 | 739 | 16.3 (15.1-17.4) | NR | NR | NR |
| *Lora-Gómez et al 2005* | Spain | Southern | Regional (Cáceres) | 1988-1999 | Retrospective | < 14 | 137 | 16.8 (14.1-19.8) | 12.7 (8.8-17.9) | 18.2 (13.7-23.8) | 19.1 (14.2-25.1) (10-13 yr) |
| *Morales-Pérez et al 2000* | Spain | Southern | Regional (Badajoz) | 1992-1996 | Retrospective and prospective | 0-29 | 186 | 16.4 (11.6-  22.6) | 9.0 (5.3-14.2) | 19.3 (13.8-26.2) | 23.4 (17.6-30.4) |
| *Mayoral et al 2018* | Spain | Southern | Regional (Asturias) | 2002-2011 | Capture-recapture method | < 40 | 436 (169 < 40 years) | 15.6 (13.33-18.13) | 9.58 (6.64-13.39) | 18.25 (14.06-23.31) | 18.78 (14.67-23.39) |
| *Nóvoa et al 2020* | Spain | Southern | Regional (Gran Canaria) | 2006-2018 | Prospective | 0-14 | 453 | 30.5 (27.7-33.4) | 22.9 (NR) | 31.5 (NR) | 36.9 (NR) |
| *Rodriguez-Escobedo et al 2022* | Spain | Southern | Regional (Asturias) | 2011-2020 | Capture-recapture method | <19 | 815 | 19.65 (17.17- 22.39) | 10.59(7.46-14.60) | 20.84 (16.64-25.76) | 26.57(21.69-32.22) |
| *Soria et al 2008* | Spain | Southern | Regional (Aragón) | 1991-2001 | Prospective | 0-14 | 303 | 16.4 (14.7-18.2) | 10.6 (8.2-13.8) | 16.6 (13.6-20.3) | 20.8 (17.5-24.6) |
| *Zorrila-Torras et al 2009* | Spain | Southern | Regional (Madrid) | 1997-2005 | Capture-recapture method | 0-14 | 1130 | 15.9 (15.0-16.8) | 11.4 (9.5-13.5) (girls)  12.8 (10.9-15.0) (boys) | 20.2 (17.6-23.0) (girls)  16.3 (14.1-18.8) (boys) | 16.1 (13.9-18.5) (girls)  18.6 (16.3-21.1) (boys) |
| *Patterson et al 2019* | Spain | Southern | Regional (Catalonia) | 1989-2013 | Capture-recapture method | 0-14 | 3447 | 13.6 (NR) (1994-1998)  13.1 (NR) (1999-2003)  12.1 (NR) (2004-2008)  16.1 (NR) (2009-2013) | 8.4 (NR) (boys)  7.9 (NR) (girls) (1994-1998)  6.4 (NR) (boys)  7.5 (NR) (girls) (1999-2003)  7.3 (NR) (boys) 7.0 (NR) (girls) (2004-2008)  11.7 (NR) (boys) 11.3 (NR) (girls) (2009-2013) | 12.4 (NR) (boys)  15.6 (NR) (girls) (1994-1998)  14.6 (NR) (boys)  14.2 (NR) (girls) (1999-2003)  12.6 (NR) (boys) 14.3 (NR) (girls) (2004-2008)  14.4 (NR) (boys) 16.7 (NR) (girls) (2009-2013) | 19.7 (NR) (boys)  17.4 (NR) (girls) (1994-1998)  19.9 (NR) (boys)  15.6 (NR) (girls) (1999-2003)  18.2 (NR) (boys) 13.3 (NR) (girls) (2004-2008)  21.6 (NR) (boys) 20.7 (NR) (girls) (2009-2013) |
| *Berhan et al 2011* | Sweden | Northern | National | 1978-2007 | Prospective | < 15 | 14721 | 29.9 (27.3-32.5) (1993-1995)  43.9 (40.7-47.3) (2005-2007) | 21.3 (17.6-25.0) (1993-1995)  25.2 (20.8-29.6) (2005-2007) | 33.7 (28.8-38.4) (1993-1995)  47.9 (41.6-54.1) (2005-2007) | 33.9 (28.9-39.1) (1993-1995)  56.5 (50.5-62.9) (2005-2007) |
| *Patterson et al 2019* | Sweden | Northern | Regional (Stockholm county) | 1989-2013 | Capture-recapture method | 0-14 | 2704 | 25.6 (NR) (1994-1998)  34.5 (NR) (1999-2003)  35.1 (NR) (2004-2008)  39.6 (NR) (2009-2013) | 18.9 (NR) (boys)  16.8 (NR) (girls) (1994-1998)  24.6 (NR) (boys)  24.7 (NR) (girls) (1999-2003)  22.6 (NR) (boys) 20.2 (NR) (girls) (2004-2008)  22.6 (NR) (boys) 21.4 (NR) (girls) (2009-2013) | 27.6 (NR) (boys)  31.8 (NR) (girls) (1994-1998)  36.4 (NR) (boys)  37.0 (NR) (girls) (1999-2003)  40.8 (NR) (boys) 40.8 (NR) (girls) (2004-2008)  38.1 (NR) (boys) 39.8 (NR) (girls) (2009-2013) | 37.1 (NR) (boys)  21.0 (NR) (girls) (1994-1998)  45.7 (NR) (boys)  38.7 (NR) (girls) (1999-2003)  50.0 (NR) (boys) 36.3 (NR) (girls) (2004-2008)  60.2 (NR) (boys) 55.7 (NR) (girls) (2009-2013) |
| *Patterson et al 2019* | Switzerland | Western | National | 1991-2013 | Capture-recapture method | 0-14 | 3020 | 8.3 (NR) (1994-1998)  11.0 (NR) (1999-2003)  13.1 (NR) (2004-2008)  13.4 (NR) (2009-2013) | 6.6 (NR) (boys)  5.3 (NR) (girls) (1994-1998)  10.7 (NR) (boys)  7.8 (NR) (girls) (1999-2003)  10.6 (NR) (boys) 10.9 (NR) (girls) (2004-2008)  9.8 (NR) (boys) 9.2 (NR) (girls) (2009-2013) | 8.7 (NR) (boys)  8.0 (NR) (girls) (1994-1998)  11.1 (NR) (boys)  10.8 (NR) (girls) (1999-2003)  13.1 (NR) (boys) 13.8 (NR) (girls) (2004-2008)  15.8 (NR) (boys) 14.1 (NR) (girls) (2009-2013) | 10.8 (NR) (boys)  10.2 (NR) (girls) (1994-1998)  13.5 (NR) (boys)  11.9 (NR) (girls) (1999-2003)  16.0 (NR) (boys) 14.0 (NR) (girls) (2004-2008)  15.6 (NR) (boys) 15.8 (NR) (girls) (2009-2013) |
| *Demirbilek et al 2013* | Turkey | Southern | National | 2010-2011 | Retrospective (data were reviewed) | 0-14 | 41 | 7.2 (NR) | 4.3 (NR) | 9.1 (NR) | 8.4 (NR) |
| *Esen et al 2020* | Turkey | Southern | Regional (Elazig) | 2009-2019 | Retrospective | < 15 | 228 | 16.7 (14.6-19.0) ^3^ | 9.6 (6.9-12.9) ^3^ | 20.7 (16.7-25.3) ^3^ | 19.4 (15.7-23.8) ^3^ |
| *Poyrazoglu et al 2018* | Turkey | Southern | Regional (Ntohewet) | 2013-2015 | Prospective | < 18 | 1773 | 8.99 (8.58-9.42) (< 18 yrs) | 6.13 (NR) | 11.68 (NR) | 11.7 (NR) |
| *Zhao et al 1999* | UK | Western | Regional (Devon and Cornwall) | 1975-1996 | Capture-recapture method | < 16 | 522 | 14.9 (13.58-16.16) ^3^ | 9.35 (7.57-11.42) | 15.81 (13.52-18.37) | 19.02 (16.44-21.51) |
| *Harron et al 2011* | UK | Western | Regional (Yorksire) | 1978-2007 | Prospective | <15 | 3912 | 18.1 (17.6-18.7) ^2^ | 11.7 (10.9-12.5) ^2^ | 18.6 (17.6-19.6) ^2^ | 23.7 (22.6-24.8) ^2^ |
| *Hayes et al 2023* | UK | Western | Regional (North East and North Cumbria) | 2012-2020 | Prospective | <14 | 943 | 21.3 (19.9-22.7) | 13.8(11.9-15.7) | 24.6(22.1-27.1) | 25.2(22.6-27.8) |
| *Raymond et al 2001* | UK | Western | Regional (Leicestershire) | 1989-1998 | Prospective | < 15 | 263 | 19.2 (12.0-29.1)^3^ (South Asians girls)  20.3 (13.0-30.3) ^3^ (South Asians boys)  17.7 (14.8-21.1) ^3^ (Whites others girls)  17.7 (14.8-20.9) ^3^ (White others boys) | 7.7 (1.6-22.6) ^3^ (South Asians girls)  4.9 (0.6-17.8) ^3^ (South Asians boys)  11.8 (0.8-16.9) ^3^ (Whites others girls)  10.5 (7.0-15.2) ^3^ (White others boys) | 27.6 (13.8-49.3) ^3^ (South Asians girls)  24.5 (11.8-45.1) ^3^ (South Asians boys)  19.8 (14.5-26.3) ^3^ (Whites others girls)  15.5 (11.0-21.1) ^3^ (White others boys) | 22.4 (9.7-44.1) ^3^ (South Asians girls)  32.8 (17.0-57.3) ^3^ (South Asians boys)  22.1 (16.4-29.0) ^3^ (Whites others girls)  27.6 (21.5-35.0) ^3^ (White others boys) |
| *Cardwell et al 2007* | UK | Western | National (Northern Ireland) | 1989-2003 | Prospective and capture-recapture method | 0-14 | 1433 | 24.7 (23.4-26.0) (1989-2003) | 16.5 (NR) (1994-1998)  27.0 (NR) (1999-2003) | 27.5 (NR) (1994-1998)  28.5 (NR) (1999-2003) | 30.5 (NR) (1994-1998)  37.5 (NR) (1999-2003) |
| *Patterson et al 2019* | UK | Western | Regional (Northern Ireland) | 1989-2013 | Capture-recapture method | 0-14 | 2652 | 24.7 (NR) (1994-1998)  29.9 (NR) (1999-2003)  34.2 (NR) (2004-2008)  34.4 (NR) (2009-2013) | 16.8 (NR) (boys)  16.6 (NR) (girls) (1994-1998)  24.8 (NR) (boys)  21.9 (NR) (girls) (1999-2003)  23.6 (NR) (boys) 20.2 (NR) (girls) (2004-2008)  19.4 (NR) (boys) 20.0 (NR) (girls) (2009-2013) | 24.5 (NR) (boys)  29.5 (NR) (girls) (1994-1998)  27.8 (NR) (boys)  30.3 (NR) (girls) (1999-2003)  32.8 (NR) (boys) 41.4 (NR) (girls) (2004-2008)  38.1 (NR) (boys) 38.8 (NR) (girls) (2009-2013) | 34.2 (NR) (boys)  26.7 (NR) (girls) (1994-1998)  38.7 (NR) (boys)  35.7 (NR) (girls) (1999-2003)  43.7 (NR) (boys) 43.4 (NR) (girls) (2004-2008)  50.2 (NR) (boys) 40.1 (NR) (girls) (2009-2013) |
| *Patterson et al 2019* | UK | Western | Regional (Oxford) | 1989-2013 | Capture-recapture method | 0-14 | 2920 | 21.7 (NR) (1994-1998)  24.0 (NR) (1999-2003)  25.2 (NR) (2004-2008)  22.8 (NR) (2009-2013) | 15.3 (NR) (boys)  14.5 (NR) (girls) (1994-1998)  15.8 (NR) (boys)  15.0 (NR) (girls) (1999-2003)  17.8 (NR) (boys) 15.3 (NR) (girls) (2004-2008)  15.9 (NR) (boys) 12.6 (NR) (girls) (2009-2013) | 20.9 (NR) (boys)  23.6 (NR) (girls) (1994-1998)  21.3 (NR) (boys)  24.1 (NR) (girls) (1999-2003)  27.9 (NR) (boys) 24.8 (NR) (girls) (2004-2008)  21.7 (NR) (boys) 23.9 (NR) (girls) (2009-2013) | 30.9 (NR) (boys)  24.9 (NR) (girls) (1994-1998)  35.0 (NR) (boys)  32.5 (NR) (girls) (1999-2003)  31.8 (NR) (boys) 33.5 (NR) (girls) (2004-2008)  32.8 (NR) (boys) 29.8 (NR) (girls) (2009-2013) |
| *Patterson et al 2019* | UK | Western | Regional (Yorkshire) | 1989-2013 | Capture-recapture method | 0-14 | 3660 | 19.7 (NR) (1994-1998)  23.5 (NR) (1999-2003)  25.5 (NR) (2004-2008)  31.0 (NR) (2009-2013) | 13.9 (NR) (boys)  13.3 (NR) (girls) (1994-1998)  16.8 (NR) (boys)  14.1 (NR) (girls) (1999-2003)  14.9 (NR) (boys) 14.9 (NR) (girls) (2004-2008)  17.5 (NR) (boys) 16.3 (NR) (girls) (2009-2013) | 17.7 (NR) (boys)  21.4 (NR) (girls) (1994-1998)  25.0 (NR) (boys)  27.0 (NR) (girls) (1999-2003)  24.0 (NR) (boys) 30.1 (NR) (girls) (2004-2008)  28.8 (NR) (boys) 35.8 (NR) (girls) (2009-2013) | 29.3 (NR) (boys)  22.3 (NR) (girls) (1994-1998)  30.1 (NR) (boys)  27.9 (NR) (girls) (1999-2003)  40.6 (NR) (boys) 28.3 (NR) (girls) (2004-2008)  49.0 (NR) (boys) 38.7 (NR) (girls) (2009-2013) |
| *Roche et al 2023* | UK | Western | National (Ireland) | 2019-2021 | Prospective; Capture-recapture | <15 | 1027 | 31.5 (28.1, 35.2) 2019 year  32.8 (29.3, 36.5) 2020 year  38.2 (34.4, 42.2)  2021 year | 15.2 (11.2, 20.2)  2019 year  16.5 (12.3, 21.7)  2020 year  24.1 (18.9, 30.3)  2021 year | 30.9 (25.4, 37.3)  2019 year  33.7 (27.9,40.4)  2020 year  45.7 (38.7,53.5)  2021 year | 47.2 (40.2, 55.0) 2019 year  46.3 (39.4,54.0)  2020 year  43.0 (36.5,50.4)  2021 year |

NR: Not reported

# Supplementary Table 3. Quality Assessment (NHLBI Cohort and Cross-sectional Studies)

| **Study** | **1** | **2** | **3** | **4** | **5** | **6** | **7** | **8** | **9** | **10** | **11** | **12** | **13** | **14** |
| --- | --- | --- | --- | --- | --- | --- | --- | --- | --- | --- | --- | --- | --- | --- |
| Rami et al, 2001 | Y | Y | Y | Y | N | NA | Y | NA | NA | NA | Y | NA | Y | NA |
| Schoeber et al 2008 | Y | Y | Y | Y | Y | NA | Y | NA | NA | NA | Y | NA | Y | NA |
| Bratina et al 2001 | Y | Y | Y | Y | Y | NA | Y | NA | NA | NA | Y | NA | Y | NA |
| Bukara-Radujkovic et al 2018 | Y | Y | Y | Y | N | NA | Y | NA | NA | NA | Y | NA | Y | NA |
| Stipancic et al 2008 | Y | Y | Y | Y | Y | NA | Y | NA | NA | NA | Y | NA | Y | NA |
| Rojnic et al 2014 | Y | Y | Y | Y | Y | NA | Y | NA | NA | NA | Y | NA | Y | NA |
| Mousa et al 2020 | Y | Y | Y | Y | Y | NA | Y | NA | NA | NA | Y | NA | Y | NA |
| Toumba et al 2007 | N | N | Y | Y | N | NA | Y | NA | NA | NA | Y | NA | Y | NA |
| Cinek et al 2000 | Y | Y | Y | Y | Y | NA | Y | NA | NA | NA | Y | NA | Y | NA |
| Cinek et al 2012 | Y | Y | Y | Y | Y | NA | Y | NA | NA | NA | Y | NA | Y | NA |
| Cinek et al 2022 | Y | Y | Y | Y | Y | NA | Y | NA | NA | NA | Y | NA | Y | NA |
| Teeäär et al 2010 | Y | N | Y | Y | Y | NA | Y | NA | NA | NA | Y | NA | Y | NA |
| Podar et al 2001 | Y | Y | Y | N | Y | NA | Y | NA | NA | NA | Y | NA | Y | NA |
| Knip,et al 2023 | Y | Y | Y | Y | Y | NA | Y | NA | NA | NA | Y | NA | Y | NA |
| Rytkönen et al 2003 | Y | Y | Y | Y | Y | NA | Y | NA | NA | NA | Y | NA | Y | NA |
| Parviainen et al 2020 | Y | Y | Y | Y | Y | NA | Y | NA | NA | NA | Y | NA | Y | NA |
| Mauny et al 2005 | Y | Y | Y | Y | Y | NA | Y | NA | NA | NA | Y | NA | Y | NA |
| Barat et al 2008 | Y | Y | Y | Y | Y | NA | Y | NA | NA | NA | Y | NA | Y | NA |
| Piffaretti et al 2019 | Y | Y | Y | Y | Y | NA | Y | NA | NA | NA | Y | NA | Y | NA |
| Baechle et al 2023 | Y | Y | Y | Y | Y | NA | Y | NA | NA | NA | Y | NA | Y | NA |
| Bendas et al 2015 | Y | Y | Y | Y | Y | NA | Y | NA | NA | NA | Y | NA | Y | NA |
| Ehehalt et al 2008 | Y | Y | Y | Y | N | NA | Y | NA | NA | NA | Y | NA | Y | NA |
| Galler et al 2010 | Y | Y | Y | Y | Y | NA | Y | NA | NA | NA | Y | NA | Y | NA |
| Rosenabuer et al 2002 | Y | Y | Y | Y | N | NA | N | NA | NA | NA | Y | NA | Y | NA |
| Weis et al 2023 | Y | Y | Y | Y | Y | NA | Y | NA | NA | NA | Y | NA | Y | NA |
| Mamoulakis et al 2018 | Y | Y | Y | Y | N | NA | Y | NA | NA | NA | Y | NA | Y | NA |
| Gyurus et al 2012 | Y | Y | Y | Y | N | NA | Y | NA | NA | NA | Y | NA | Y | NA |
| Bruno et al 2010 | Y | N | Y | Y | Y | NA | Y | NA | NA | NA | Y | NA | Y | NA |
| Fortunato et al 2016 | Y | Y | Y | Y | Y | NA | Y | NA | NA | NA | Y | NA | Y | NA |
| Giorda et al 2023 | Y | Y | Y | Y | Y | NA | Y | NA | NA | NA | Y | NA | Y | NA |
| Gesuita el al 2023 | Y | Y | Y | Y | Y | NA | Y | NA | NA | NA | Y | NA | Y | NA |
| Passanisi et al 2022 | Y | Y | Y | Y | Y | NA | Y | NA | NA | NA | Y | NA | Y | NA |
| Roche et al 2002 | N | Y | Y | Y | N | NA | Y | NA | NA | NA | Y | NA | Y | NA |
| Roche et al 2016 | Y | N | Y | Y | Y | NA | Y | NA | NA | NA | Y | NA | Y | NA |
| Urbonaite et al 2002 | Y | N | Y | Y | Y | NA | Y | NA | NA | NA | Y | NA | Y | NA |
| Formosa et al 2012 | Y | Y | Y | Y | Y | NA | Y | NA | NA | NA | Y | NA | Y | NA |
| Raicevic et al 2022 | y | y | y | y | y | NA | y | NA | NA | NA | Y | NA | Y | NA |
| Samardzic et al 2011 | Y | Y | Y | Y | Y | NA | Y | NA | NA | NA | Y | NA | Y | NA |
| Spaans et al 2015 | Y | Y | Y | Y | Y | NA | N | NA | NA | NA | Y | NA | Y | NA |
| Fazeli Farsani et al 2016 | Y | Y | Y | Y | Y | NA | Y | NA | NA | NA | Y | NA | Y | NA |
| Aamodt et al 2007 | Y | Y | Y | Y | Y | NA | Y | NA | NA | NA | Y | NA | Y | NA |
| Skrivarhaug et al 2014 | Y | Y | Y | Y | Y | NA | Y | NA | NA | NA | Y | NA | Y | NA |
| Jarosz-Chobot et al 2011 | Y | Y | Y | Y | Y | NA | Y | NA | NA | NA | Y | NA | Y | NA |
| Szalecki et al 2016 | Y | Y | Y | Y | Y | NA | Y | NA | NA | NA | Y | NA | Y | NA |
| Chobot et al 2017 | Y | Y | Y | Y | Y | NA | Y | NA | NA | NA | Y | NA | Y | NA |
| Serban et al 2015 | Y | Y | Y | Y | Y | NA | Y | NA | NA | NA | Y | NA | Y | NA |
| Vlad et al 2018 | Y | Y | Y | Y | Y | NA | Y | NA | NA | NA | Y | NA | Y | NA |
| Sipetic et al 2013 | Y | Y | Y | Y | Y | NA | Y | NA | NA | NA | Y | NA | Y | NA |
| Vorgucˇin et al 2022 | Y | Y | Y | Y | Y | NA | Y | NA | NA | NA | Y | NA | Y | NA |
| Bahíllo et al 2007 | Y | Y | Y | Y | Y |  | N | NA | NA | NA | Y | NA | Y | NA |
| Fernández-Ramos et al. 2017 | Y | Y | Y | Y | Y | NA | Y | NA | NA | NA | Y | NA | Y | NA |
| Forga-Llenas et al 2018 | Y | Y | Y | Y | N | NA | Y | NA | NA | NA | Y | NA | Y | NA |
| Gimeno-Benítez et al 2014 | Y | Y | Y | Y | N | NA | Y | NA | NA | NA | Y | NA | Y | NA |
| Giralt-Muiña et al 2012 | Y | Y | Y | Y | Y | NA | N | NA | NA | NA | Y | NA | Y | NA |
| Goñi-Iriarte et al. 2023 | Y | Y | Y | Y | Y | NA | N | NA | NA | NA | Y | NA | Y | NA |
| Hernández-Herrero et al 2021 | Y | Y | Y | Y | Y | NA | N | NA | NA | NA | Y | NA | Y | NA |
| López-Siguero et al 2002 | Y | Y | Y | Y | N | NA | Y | NA | NA | NA | Y | NA | Y | NA |
| Lora-Gómez et al 2005 | Y | Y | Y | Y | N | NA | Y | NA | NA | NA | Y | NA | Y | NA |
| Morales-Pérez et al 2000 | Y | Y | Y | Y | Y | NA | Y | NA | NA | NA | Y | NA | Y | NA |
| Mayoral et al 2018 | Y | Y | Y | Y | Y | NA | Y | NA | NA | NA | Y | NA | Y | NA |
| Nóvoa et al 2020 | Y | Y | Y | Y | Y | NA | Y | NA | NA | NA | Y | NA | Y | NA |
| Rodriguez-Escobedo et al 2022 | Y | Y | Y | Y | Y | NA | Y | NA | NA | NA | Y | NA | Y | NA |
| Soria et al 2008 | Y | Y | Y | Y | Y | NA | Y | NA | NA | NA | Y | NA | Y | NA |
| Zorrila-Torras et al 2009 | Y | Y | Y | Y | Y | NA | Y | NA | NA | NA | Y | NA | Y | NA |
| Berhan et al 2011 | Y | Y | Y | Y | Y | NA | Y | NA | NA | NA | Y | NA | Y | NA |
| Demirbilek et al 2013 | Y | Y | Y | Y | Y | NA | N | NA | NA | NA | Y | NA | Y | NA |
| Esen et al 2020 | Y | Y | Y | Y | Y | NA | Y | NA | NA | NA | Y | NA | Y | NA |
| Poyrazoglu et al 2018 | Y | Y | Y | Y | Y | NA | N | NA | NA | NA | Y | NA | Y | NA |
| Zhao et al 1999 | Y | Y | Y | Y | Y | NA | Y | NA | NA | NA | Y | NA | Y | NA |
| Harron et al 2011 | Y | Y | Y | Y | Y | NA | Y | NA | NA | NA | Y | NA | Y | NA |
| Hayes et al 2023 | Y | Y | Y | Y | Y | NA | Y | NA | NA | NA | Y | NA | Y | NA |
| Raymond et al 2001 | Y | Y | Y | Y | Y | NA | Y | NA | NA | NA | Y | NA | Y | NA |
| Cardwell et al 2007 | Y | Y | Y | Y | Y | NA | Y | NA | NA | NA | Y | NA | Y | NA |
| C. Patterson et al 2019 | Y | Y | Y | Y | Y | NA | Y | NA | NA | NA | Y | NA | Y | NA |
| Roche et al 2023 | Y | Y | Y | Y | Y | NA | Y | NA | NA | NA | Y | NA | Y | NA |

CD = Cannot determine, N = No, NA = Not applicable, NR = Not reported, Y = Yes

1. Was the research question or objective in this paper clearly stated?

2. Was the study population clearly specified and defined?

3. Was the participation rate of eligible persons at least 50%?

4. Were all the subjects selected or recruited from the same or similar populations (including the same time period)? Were inclusion and exclusion criteria for being in the study prespecified and applied uniformly to all participants?

5. Was a sample size justification, power description, or variance and effect estimates provided?

6. For the analyses in this paper, were the exposure(s) of interest measured prior to the outcome(s) being measured?

7. Was the timeframe sufficient so that one could reasonably expect to see an association between exposure and outcome if it existed?

8. For exposures that can vary in amount or level, did the study examine different levels of the exposure as related to the outcome (e.g., categories of exposure, or exposure measured as continuous variable

9. Were the exposure measures (independent variables) clearly defined, valid, reliable, and implemented consistently across all study participants?

10. Was the exposure(s) assessed more than once over time?

11. Were the outcome measures (dependent variables) clearly defined, valid, reliable, and implemented consistently across all study participants?

12. Were the outcome assessors blinded to the exposure status of participants?

13. Was loss to follow-up after baseline 20% or less?

14. Were key potential confounding variables measured and adjusted statistically for their impact on the relationship between exposure(s) and outcome(s)

# Supplementary Table 4. Trends in the incidence rates (pool estimate (95% confidence interval) of childhood diabetes type 1 by sex and age group for 32 countries of Europe

|  | | **1994-2003** | | | | | | **2004-2012** | | | | | | **2013-2022** | | | | | | **Δ diabetes 1994-2022** | | | | | | | |
| --- | --- | --- | --- | --- | --- | --- | --- | --- | --- | --- | --- | --- | --- | --- | --- | --- | --- | --- | --- | --- | --- | --- | --- | --- | --- | --- | --- |
| Country | | 0-4 yr | | 5-9 yr | | 10-14 yr | | 0-4 yr | | 5-9 yr | | 10-14yr | | 0-4 yr | | 5-9 yr | | 10-14 yr | | 0-4 yr | | 5-9 yr | | 10-14 yr | | |  |
| ***Austria*** | |  | |  | |  | |  | |  | |  | |  | |  | |  | |  | |  | |  | | |  |
| Boys | | 7.33  (2.15-12.51) ^2^ | | 11.99  (11.02-12.97) ^1^ | | 12.99  (9.30-16.67) ^2^ | | 14.3  (13.3-15.3) ^1^ | | 20.10  (19.10-21.10) ^1^ | | 24.6  (23.6-25.60) ^1^ | | - | | - | | - | | +6.97 ^a^ | | +8.11 ^a^ | | +11.61 ^a^ | | |  |
| Girls | | 7.28  (6.31-8.25) ^1^ | | 12.45  (11.48-13.42) ^1^ | | 13.02  (12.04-14.01) ^1^ | | 12.35  (11.35-13.35) ^1^ | | 18.20  (17.20-19.20) ^1^ | | 21.35  (20.35-22.35) ^1^ | | - | | - | | - | | +5.07 ^a^ | | + 5.75 ^a^ | | + 8.33 ^a^ | | |  |
| Overall | | 7.36  (4.61-10.12) ^2^ | | 12.17  (11.21-13.13) ^1^ | | 13.51  (12.54-14.47) ^1^ | | 13.32  (12.32-14.32) ^1^ | | 12.37  (11.37-13.37) ^1^ | | 20.47  (19.47-21.47) ^1^ | | - | | - | | - | | +5.96 ^a^ | | +0.20 | | +6.96 ^a^ | | |  |
| ***Belgium*** | |  | |  | |  | |  | |  | |  | |  | |  | |  | |  | |  | |  | | |  |
| Boys | | 10.7  (8.7-12.70) ^1^ | | 17.95  (15.95-19.95) ^1^ | | 19.4  (17.4-21.4) ^1^ | | 10.65  (8.65-12.65) ^1^ | | 19.0  (17.0-21.0) ^1^ | | 23.6  (21.6-25.6) ^1^ | | - | | - | | - | | -0.05 | | +1.05 | | +4.20 ^a^ | | |  |
| Girls | | 10.7  (8.7-12.70) ^1^ | | 17.95  (15.95-19.95) ^1^ | | 16.85  (14.85-18.85) ^1^ | | 11.05  (9.05-13.05) ^1^ | | 18.3  (16.3-20.3) ^1^ | | 20.55  (18.55-22.55) ^1^ | | - | | - | | - | | +0.35 | | +0.35 | | +3.70 | | |  |
| Overall | | 10.15  (8.15-12.15) ^1^ | | 15.87  (13.87-17.87) ^1^ | | 18.12  (16.12-20.12) ^1^ | | 10.85  (8.85-12.85) ^1^ | | 19.1  (17.10-21.1) ^1^ | | 22.07  (20.07-24.07) ^1^ | | - | | - | | - | | +0.70 | | +3.23 | | +3.95 | | |  |
| ***Bosnia and Herzegovina*** | |  | |  | |  | |  | |  | |  | |  | |  | |  | |  | |  | |  | | |  |
| Boys | | 1.04  (0.00-2.08) ^1^ | | 4.59  (1.60-7.58) ^1^ | | 5.07  (1.70-8.44) ^1^ | | - | | - | | - | | - | | - | | - | | - | | - | | - | | |  |
| Girls | | 0.13  (0.00-0.26) ^1^ | | 4.78  (3.70-5.86) ^1^ | | 5.26  (2.60-7.92) ^1^ | | - | | - | | - | | - | | - | | - | | - | | - | | - | | |  |
| Overall | | 2.19  (1.16-5.55) ^2^ | | 7.04  (1.61-12.48) ^2^ | | 4.92  (3.04-6.80) ^1^ | | 5.70  (3.59-7.81) ^1^ | | 13.90  (10.57-17.23) ^1^ | | 13.50  (10.36-16.64) ^1^ | | 9.68  (4.29-15.07) ^2^ | | 16.80  (13.31-20.29) ^2^ | | 22.70  (17.72-27.68)^2^ | | +7.49 | | +9.76 ^b^ | | +17.78 ^b,c^ | | |  |
| ***Croatia*** | |  | |  | |  | |  | |  | |  | |  | |  | |  | |  | |  | |  | | |  |
| Boys | | 8.04  (5.09-10.99) ^2^ | | 15.04  (3.2-26.88) ^2^ | | 11.91  (10.97-12.85) ^1^ | | 14.18  (12.45-15.90) ^2^ | | 17.02  (15.41-18.63) ^1^ | | 18.08  (16.45-19.71) ^2^ | | - | | - | | - | | +6.14 ^a^ | | +1.98 | | +6.17 ^a^ | | |  |
| Girls | | 5.96  (3.86-8.05) ^2^ | | 12.64  (8.86-16.42) ^2^ | | 11.80  (8.17-15.42) ^2^ | | 12.73  (11.16-14.29) ^1^ | | 22.38  (20.69-24.07) ^1^ | | 22.61  (12.96-32.25) ^2^ | | - | | - | | - | | +6.77 ^a^ | | +9.74 ^a^ | | +10.80 | | |  |
| Overall | | 6.87  (4.39-9.34) ^2^ | | 13.75  (5.91-21.59) ^2^ | | 11.55  (10.48-12.63) ^1^ | | 13.41  (12.13-14.70) ^1^ | | 21.01  (16.57-25.46) ^2^ | | 21.58  (18.48-24.68) ^2^ | | - | | - | | - | | +6.54 ^a^ | | +7.26 | | +10.03 ^a^ | | |  |
|  | **1994-2003** | | | | | | **2004-2012** | | | | | | **2013-2022** | | | | | | **Δ diabetes 1994-2022** | | | | | | |  |  |
| Country | 0-4 yr | | 5-9 yr | | 10-14 yr | | 0-4 yr | | 5-9 yr | | 10-14yr | | 0-4 yr | | 5-9 yr | | 10-14 yr | | 0-4 yr | | 5-9 yr | | 10-14 yr | |  |  |  |
| ***Cyprus*** |  | |  | |  | |  | |  | |  | |  | |  | |  | |  | |  | |  | |  |  |  |
| Boys | - | | - | | - | | - | | - | | - | | - | | - | | - | | - | | - | | - | |  |  |  |
| Girls | - | | - | | - | | - | | - | | - | | - | | - | | - | | - | | - | | - | |  |  |  |
| Overall | - | | - | | - | | 5.75  (3.02-8.48)^1^ | | 15.40  (10.30-20.50)^1^ | | 17.60  (12.00-23.20)^1^ | | - | | - | | - | | - | | - | | - | |  |  |  |
| ***Crech Republic*** |  | |  | |  | |  | |  | |  | |  | |  | |  | |  | |  | |  | |  |  |  |
| Boys | 9.21  (5.49-12.92)^2^ | | 13.12  (9.65-16.59)^2^ | | 16.07  (12.37-19.76)^2^ | | 14.61  (13.87-15.34)^1^ | | 22.97  (22.21-23.73)^1^ | | 26.51  (25.75-27.27)^1^ | | - | | - | | - | | +5.40 ^a^ | | +9.85 ^a^ | | +10.44 ^a^ | |  |  |  |
| Girls | 8.15  (4.94-11.36)^2^ | | 14.76  (10.59-18.93)^2^ | | 16.42  (15.10-17.75)^1^ | | 12.83  (12.18-13.48)^1^ | | 23.31  (23.02-23.61)^1^ | | 21.90  (20.02-23.77)^2^ | | - | | - | | - | | +4.68 ^a^ | | +8.55 ^a^ | | +5.48 ^a^ | |  |  |  |
| Overall | 8.63  (5.07-12.19)^2^ | | 13.23  (8.62-17.83)^2^ | | 16.23  (13.70-18.77)^2^ | | 13.19  (11.68-14.71)^2^ | | 22.99  (22.27-23.72)^1^ | | 23.92  (22.68-25.58)^2^ | | - | | - | | - | | +4.56 | | +9.76 ^a^ | | +7.69 ^a^ | |  |  |  |
| ***Denmark*** |  | |  | |  | |  | |  | |  | |  | |  | |  | |  | |  | |  | |  |  |  |
| Boys | 11.05  (10.05-12.05)^1^ | | 18.50  (17.5-19.5)^1^ | | 26.60  (25.60-27.60)^1^ | | 15.30  (14.30-16.30)^1^ | | 25.85  (24.85-26.85)^1^ | | 38.00  (37.00-39.00)^1^ | | - | | - | | - | | +4.25 ^a^ | | +7.35 ^a^ | | +11.40 ^a^ | |  |  |  |
| Girls | 13.15  (12.15-14.15)^1^ | | 20.65  (19.65-21.65)^1^ | | 26.60  (25.60-27.60)^1^ | | 13.35  (12.35-14.35)^1^ | | 28.65  (27.65-29.65)^1^ | | 35.40  (34.40-36.40)^1^ | | - | | - | | - | | +0.20 | | +8.00 ^a^ | | +8.80 ^a^ | |  |  |  |
| Overall | 12.10  (11.10-13.10)^1^ | | 19.57  (18.57-20.57)^1^ | | 26.60  (25.60-27.60)^1^ | | 14.32  (13.32-15.32)^1^ | | 27.25  (26.25-28.25)^1^ | | 36.70  (35.70-37.70)^1^ | | - | | - | | - | | +2.22 ^a^ | | +7.68 ^a^ | | +10.11 ^a^ | |  |  |  |
| ***Estonia*** |  | |  | |  | |  | |  | |  | |  | |  | |  | |  | |  | |  | |  |  |  |
| Boys | 10.3  (6.94-13.66)^1^ | | 11.70  (8.52-14.88)^1^ | | 16.5  (12.74-20.26)^1^ | | - | | - | | - | | - | | - | | - | | - | | - | | - | |  |  |  |
| Girls | 7.50  (4.56-10.44)^1^ | | 9.60  (6.66-12.54)^1^ | | 16.8  (12.95-20.65)^1^ | | - | | - | | - | | - | | - | | - | | - | | - | | - | |  |  |  |
| Overall | 12.20  (5.54-18.86)^2^ | | 15.81  (5.42-26.19)^2^ | | 15.48  (13.65-17.30)^1^ | | - | | - | | - | | - | | - | | - | | - | | - | | - | |  |  |  |
| ***Finland*** |  | |  | |  | |  | |  | |  | |  | |  | |  | |  | |  | |  | |  |  |  |
| Boys | 38.25  (24.78-51.72)^2^ | | 52.85  (37.86-67.84)^2^ | | 51.57  (47.84-55.30)^2^ | | 50.40  (44.37-56.44)^2^ | | 67.17  (61.94-72.40)^2^ | | 65.49  (61.94-70.05)^1^ | | 45.64  (31.39-59.88)^2^ | | 69.08  (60.95-72.21)^2^ | | 69.55  (64.13-76.36)^2^ | | +7.39 | | +16.43 | | +17.98 ^a,b^ | |  |  |  |
| Girls | 38.00  (27.22-48.78)^2^ | | 45.75  (44.78-46.71)^1^ | | 47.71  (31.39-64.02)^2^ | | 45.63  (41.19-50.06)^2^ | | 59.10  (53.42-64.78)^2^ | | 45.91  (42.90-48.93)^1^ | | 39.70  (35.30-44.10)^1^ | | 56.14  (46.08-56.19)^2^ | | 48.33  (43.72-52.95)^1^ | | +1.70 | | +10.39 ^a,b^ | | +0.62 | |  |  |  |
| Overall | 38.05  (25.88-50.22)^2^ | | 48.72  (40.12-57.31)^2^ | | 49.43  (39.32-59.54)^2^ | | 48.16  (43.04-53.27)^2^ | | 63.25  (58.23-68.26)^2^ | | 58.88  (53.52-58.24)^1^ | | 42.58  (35.12-50.05)^1^ | | 63.84  (52.70-74.97)^2^ | | 59.16  (55.56-62.76-)^1^ | | +4.53 | | +15.12 ^a^ | | +9.73 | |  |  |  |

|  | **1994-2003** | | | **2004-2012** | | | **2013-2022** | | | **Δ diabetes 1994-2022** | | | |
| --- | --- | --- | --- | --- | --- | --- | --- | --- | --- | --- | --- | --- | --- |
| Country | 0-4 yr | 5-9 yr | 10-14 yr | 0-4 yr | 5-9 yr | 10-14yr | 0-4 yr | 5-9 yr | 10-14 yr | 0-4 yr | 5-9 yr | 10-14 yr |  |
| ***France*** |  |  |  |  |  |  |  |  |  |  |  |  |  |
| Boys | - | - | - | - | - | - | - | - | - | - | - | - |  |
| Girls | - | - | - | - | - | - | - | - | - | - | - | - |  |
| Overall | 6.03  (4.76-7.53)^1^ | 6.45  (5.23-7.88)^1^ | 8.56  (7.18-10.13)^1^ | - | - | - | 14.20  (12.9-15.4)^1^ | 19.40  (18.1-20.8)^1^ | 23.10  (21.7-24.6)^1^ | +8.17 ^b^ | +12.95 ^b^ | +14.54 ^b^ |  |
| ***Germany*** |  |  |  |  |  |  |  |  |  |  |  |  |  |
| Boys | 12.74  (8.49-16.98)^2^ | 15.83  (12.40-19.27)^2^ | 18.03  (17.35-18.71)^1^ | 15.60  (15.3-15.9)^1^ | 24.75  (24.05-25.45)^1^ | 29.55  (28.85-30.25)^1^ | - | - | - | +2.86 | +8.92 | +11.52 ^a^ |  |
| Girls | 9.92  (9.25-10.59)^1^ | 17.42  (13.40-21.44)^2^ | 16.77  (16.10-17.45)^1^ | 16.70  (16.00-17.40)^1^ | 25.05  (24.35-25.75)^1^ | 21.63  (20.23-23.04)^2^ | - | - | - | +6.78 ^a^ | +7.65 ^a^ | +4.86 ^a^ |  |
| Overall | 12.65  (9.87-14.66)^2^ | 18.58  (15.73-21.44)^2^ | 19.87  (17.57-22.18)^2^ | 17.08  (16.36-17.81)^2^ | 24.97  (23.78-26.16)^2^ | 25.78  (24.47-27.08)^2^ | 19.73  (16.70-22.77)^2^ | 33.97  (30.25-37.69)^2^ | 24.40  (23.26-25.53)^1^ | +7.08 ^a,b^ | +15.39 ^a,b,c^ | +4.53 ^a,b^ |  |
| ***Greece*** |  |  |  |  |  |  |  |  |  |  |  |  |  |
| Boys | - | - | - | - | - | - | - | - | - | - | - | - |  |
| Girls | - | - | - | - | - | - | - | - | - | - | - | - |  |
| Overall | 5.08  (3.02-7.15)^1^ | 8.75  (4.38-13.12)^2^ | 8.70  (6.09-11.32)^1^ | 7.14  (4.71-9.57)^1^ | 13.32  (9.00-15.64)^1^ | 10.96  (8.03-13.89)^1^ | 9.10  (5.20-14.90)^1^ | 18.60  (12.8-26.3)^1^ | 19.80  (13.70-27.79)^1^ | +4.02 | +9.85 | +11.10^b^ |  |
| ***Hungary*** |  |  |  |  |  |  |  |  |  |  |  |  |  |
| Boys | 8.15  (7.60-8.69)^1^ | 11.53  (10.98-12.08)^1^ | 14.43  (12.64-16.22)^2^ | 14.49  (13.91-15.07)^1^ | 21.77  (21.18-22.35)^1^ | 23.96  (23.11-24.81)^1^ | - | - | - | +6.34 ^a^ | +10.24 ^a^ | +9.53 ^a^ |  |
| Girls | 8.31  (7.75-8.88)^1^ | 12.94  (12.38-13.49)^1^ | 13.70  (13.14-14.26)^1^ | 13.99  (13.41-14.57)^1^ | 20.85  (20.27-21.44)^1^ | 18.97  (17.15-20.79)^2^ | - | - | - | +5.68 ^a^ | +7.91 | +5.27 ^a^ |  |
| Overall | 8.20  (7.71-8.69)^1^ | 12.24  (11.73-12.76)^1^ | 14.08  (13.56-14.60)^1^ | 14.23  (13.67-14.79)^1^ | 21.27  (20.70-21.84)^1^ | 21.21  (19.49-22.92)^2^ | - | - | - | +6.02 ^a^ | +9.03 ^a^ | +7.13 ^a^ |  |
| **Ireland** |  |  |  |  |  |  |  |  |  |  |  |  |  |
| Boys | 9.30  (4.80-16.30)^1^ | 20.40  (13.70-29.30)^1^ | 19.60  (13.40-27.70)^1^ | 18.58  (17.23-19.93)^1^ | 29.65  (26.16-33.14)^1^ | 35.44  (30.65-40.23)^2^ | 20.38  (16.78-32.98-)^1^ | 34.74  (24.06-45.42)^2^ | 43.88  (38.48-49.28)^1^ | +11.08 ^a,b^ | +14.34 | +24.28 ^a,b^ |  |
| Girls | 12.40  (6.90-20.40)^1^ | 22.10  (14.90-31.60)^1^ | 14.20  (8.90-21.60)^1^ | 16.00  (12.30-19.71)^2^ | 29.44  (28.05-30.82)^1^ | 33.39  (29.46-37.31)^1^ | 19.96  (10.66-29.26)^2^ | 34.89  (26.70-43.08)^2^ | 39.08  (31.04-47.13)^2^ | +7.56 | +12.79 | +24.88 ^a,b^ |  |
| Overall | 10.80  (7.10-15.70)^1^ | 21.30  (16.20-27.40)^1^ | 17.00  (12.80-22.20)^1^ | 18.15  (16.65-19.65)^1^ | 29.42  (27.92-30.92)^1^ | 34.90  (33.40-36.40)^1^ | 19.48  (10.77-28.20)^2^ | 38.13  (23.63-52.63)^2^ | 44.94  (40.18-49.71) | +8.68 | +8.12 ^a^ | +17.90 ^a^ |  |

|  | **1994-2003** | | | **2004-2012** | | | **2013-2022** | | | **Δ diabetes 1994-2022** | | | |
| --- | --- | --- | --- | --- | --- | --- | --- | --- | --- | --- | --- | --- | --- |
| Country | 0-4 yr | 5-9 yr | 10-14 yr | 0-4 yr | 5-9 yr | 10-14yr | 0-4 yr | 5-9 yr | 10-14 yr | 0-4 yr | 5-9 yr | 10-14 yr |  |
| *I****taly*** |  |  |  |  |  |  |  |  |  |  |  |  |  |
| Boys | 9.65  (7.85-11.45)^1^ | 11.90  (10.10-13.70)^1^ | 15.65  (13.85-17.45)^1^ | 13.09  (6.36-19.81)^2^ | 19.27  (10.46-38.09)^2^ | 21.60  (5.83-37.38)^2^ | - | - | - | +3.44 | +7.37 | +5.95 |  |
| Girls | 10.30  (9.50-11.10)^1^ | 14.55  (12.75-16.35)^1^ | 11.45  (9.65-13.25)^1^ | 13.28  (6.37-20.18)^2^ | 18.50  (10.23-36.67)^2^ | 19.40  (5.59-33.22)^2^ | - | - | - | +2.98 | +3.95 | +7.95 |  |
| Overall | 9.31  (4.81-13.81)^2^ | 13.49  (12.78-14.20)^1^ | 14.31  (12.60-16.03)^2^ | 13.24  (6.40-20.07)^2^ | 21.46  (5.44-37.48)^2^ | 20.59  (15.79-35.39)^2^ | 14.65  (9.65-19.65)^2^ | 23.96  (19.92-28.01)^1^ | 26.30  (20.74-31.86)^2^ | +5.34 | +10.47.^b^ | +11.99 ^b^ ^a^ |  |
| ***Latvia*** |  |  |  |  |  |  |  |  |  |  |  |  |  |
| Boys | 4.30  (2.90-5.70)^1^ | 6.70  (5.00-8.40)^1^ | 9.80  (7.70-11.90)^1^ | - | - | - | - | - | - | - | - | - |  |
| Girls | 4.30  (2.80-5.80)^1^ | 8.70  (6.70-10.70)^1^ | 9.30  (7.20-11.40)^1^ | - | - | - | - | - | - | - | - | - |  |
| Overall | 4.30  (3.15-5.45)^1^ | 7.70  (6.30-9.10)^1^ | 9.50  (7.93-11.03)^1^ | - | - | - | - | - | - | - | - | - |  |
| ***Lithuania*** |  |  |  |  |  |  |  |  |  |  |  |  |  |
| Boys | 5.00  (3.53-6.47)^2^ | 6.56  (5.97-7.16)^2^ | 11.99  (10.27-13.70)^2^ | 11.75  (10.80-12.70)^1^ | 13.80  (12.90-14.70)^1^ | 24.00  (23.10-24.90)^1^ | - | - | - | - | - | - |  |
| Girls | 4.46  (2.86-6.05)^2^ | 9.98  (8.75-11.21)^2^ | 11.38  (10.09-12.67)^2^ | 10.55  (9.65-11.45)^1^ | 22.40  (21.50-23.30)^1^ | 19.90  (19.00-20.80)^1^ | - | - | - | - | - | - |  |
| Overall | 5.91  (3.75-8.07)^2^ | 8.19  (7.51-8.85)^1^ | 11.59  (10.11-13.06)^2^ | 11.15  (10.25-12.05)^1^ | 18.10  (17.20-19.00)^1^ | 21.95  (21.05-22.85)^1^ | - | - | - | - | - | - |  |
| ***Luxembourg*** |  |  |  |  |  |  |  |  |  |  |  |  |  |
| Boys | 6.30  (3.50-9.10)^1^ | 13.25  (10.45-16.05)^1^ | 20.25  (17.45-23.05)^1^ | 10.15  (7.35-12.95)^1^ | 19.75  (16.95-22.55)^1^ | 28.65  (25.85-31.45)^1^ | - | - | - | +3.85 | +6.50 ^a^ | +8.40 ^a^ |  |
| Girls | 8.85  (6.05-11.65)^1^ | 18.15  (15.35-20.95)^1^ | 17.4  (14.6-20.20)^1^ | 13.10  (10.30-15.90)^1^ | 15.35  (12.55-18.15)^1^ | 24.00  (21.20-26.80)^1^ | - | - | - | +4.25 | -2.80 | +6.50 ^a^ |  |
| Overall | 7.57  (4.77-10.37)^1^ | 15.70  (12.90-18.50)^1^ | 18.82  (16.00-21.64)^1^ | 11.62  (8.82-14.42)^1^ | 17.55  (14.75-20.35)^1^ | 26.32  (23.52-29.12)^1^ | - | - | - | +4.05 | +1.85 | +7.50 ^a^ |  |

|  | **1994-2003** | | | **2004-2012** | | | **2013-2022** | | | **Δ diabetes 1994-2022** | | | |
| --- | --- | --- | --- | --- | --- | --- | --- | --- | --- | --- | --- | --- | --- |
| Country | 0-4 yr | 5-9 yr | 10-14 yr | 0-4 yr | 5-9 yr | 10-14yr | 0-4 yr | 5-9 yr | 10-14 yr | 0-4 yr | 5-9 yr | 10-14 yr |  |
| ***Malta*** |  |  |  |  |  |  |  |  |  |  |  |  |  |
| Boys | - | - | - | - | - | - | - | - | - | - | - | - |  |
| Girls | - | - | - | - | - | - | - | - | - | - | - | - |  |
| Overall | - | - | - | 21.70  (12.63-30.77)^1^ | 32.22  (21.84-42.60)^1^ | 17.68  (10.29-25.07)^1^ | - | - | - | - | - | - |  |
| ***Montenegro*** |  |  |  |  |  |  |  |  |  |  |  |  |  |
| Boys | 9.16  (7.11-11.21)^1^ | 9.92  (8.77-11.07)^1^ | 16.98  (14.85-19.11)^1^ | 14.52  (12.40-16.63)^1^ | 20.09  (17.95-22.23)^1^ | 22.27  (20.14-24.40)^1^ | - | - | - | +5.36 ^a^ | +10.17 ^a^ | +5.29 ^a^ |  |
| Girls | 10.57  (8.60-12.54)^1^ | 14.51  (12.40-16.63)^1^ | 12.76  (10.62-14.90)^1^ | 10.70  (8.71-12.69)^1^ | 24.98  (22.83-27.13)^1^ | 15.21  (13.31-17.11)^1^ | - | - | - | +0.13 | +10.47 | +2.45 |  |
| Overall | 9.30  (7.49-11.11)^1^ | 11.89  (9.96-13.82)^1^ | 14.84  (12.72-16.96)^1^ | 12.60  (10.55-14.65)^1^ | 21.71  (19.81-23.61)^1^ | 18.79  (16.79-20.80)^1^ | - | - | - | +3.30 | +9.82 ^a^ | +3.95 |  |
| ***North Macedonia*** |  |  |  |  |  |  |  |  |  |  |  |  |  |
| Boys | 2.55  (1.75-3.35)^1^ | 5.45  (4.65-6.25)^1^ | 7.05  (6.25-7.85)^1^ | 5.40  (4.60-6.20)^1^ | 8.35  (7.55-9.15)^1^ | 7.90  (7.10-8.70)^1^ | - | - | - | +2.85 | +2.90 ^a^ | +0.85 |  |
| Girls | 1.85  (1.00-2.70)^1^ | 6.65  (5.85-7.45)^1^ | 6.15  (5.35-6.95)^1^ | 5.20  (4.4-6.00)^1^ | 9.15  (8.35-9.95)^1^ | 7.05  (6.25-7.85)^1^ | - | - | - | +3.35 | +2.50 ^a^ | +0.90 |  |
| Overall | 2.20  (1.4-3.00)^1^ | 6.05  (5.25-6.85)^1^ | 6.60  (5.80-7.40)^1^ | 6.12  (4.72-7.52)^1^ | 8.75  (7.95-9.55)^1^ | 7.47  (6.60-8.34)^1^ | - | - | - | +3.92 ^a^ | +2.70 ^a^ | +0.87 |  |
| ***Norway*** |  |  |  |  |  |  |  |  |  |  |  |  |  |
| Boys | 16.70  (15.70-17.70)^1^ | 31.15  (30.35-31.95)^1^ | 37.75  (36.75-38.75)^1^ | 21.16  (20.24-22.08)^1^ | 30.41  (33.46-35.35)^1^ | 47.88  (46.92-48.84)^1^ | - | - | - | +4.46 ^a^ | -0.74 | +10.13^a^ |  |
| Girls | 16.35  (15.35-17.35)^1^ | 30.30  (29.30-31.30)^1^ | 30.20  (29.20-31.20)^1^ | 18.11  (17.20-19.01)^1^ | 38.20  (37.24-39.15)^1^ | 39.85  (38.89-40.80)^1^ | - | - | - | +1.76 | +7.90 ^a^ | +9.65 ^a^ |  |
| Overall | 16.52  (15.52-17.52)^1^ | 30.82  (29.82-31.82)^1^ | 33.97  (32.97-34.97)^1^ | 19.57  (18.73-20.42)^1^ | 36.21  (35.30-37.12)^1^ | 43.60  (42.06-45.14)^1^ | - | - | - | +3.05 ^a^ | +5.39 ^a^ | +9.63 ^a^ |  |

|  | **1994-2003** | | | **2004-2012** | | | **2013-20212** | | | **Δ diabetes 1994-2022** | | | |
| --- | --- | --- | --- | --- | --- | --- | --- | --- | --- | --- | --- | --- | --- |
| Country | 0-4 yr | 5-9 yr | 10-14 yr | 0-4 yr | 5-9 yr | 10-14yr | 0-4 yr | 5-9 yr | 10-14 yr | 0-4 yr | 5-9 yr | 10-14 yr |  |
| ***Poland*** |  |  |  |  |  |  |  |  |  |  |  |  |  |
| Boys | 6.48  (5.76-7.20)^1^ | 10.55  (9.76-11.33)^1^ | 13.83  (12.14-15.52)^2^ | 12.25  (11.43-13.06)^1^ | 18.24  (14.66-21.83)^2^ | 22.01  (19.41-24.60)^2^ | 15.66  (12.76-18.55)^1^ | 23.81  (20.21-27.40)^1^ | 30.16  (26.02-34.29)^1^ | +9.18 ^a,b,^ | +13.26 ^a,b^ | +16.33^a,b,c^ |  |
| Girls | 5.56  (4.86-6.26)^1^ | 12.04  (11.84-12.23)^1^ | 13.14  (11.86-14.42)^2^ | 13.49  (12.65-14.32)^1^ | 21.73  (20.87-22.59)^1^ | 19.26  (18.41-20.11)^1^ | 14.78  (11.89-17.66)^1^ | 21.46  (17.97-24.96)^1^ | 23.13  (19.41-26.84)^1^ | +9.22^a,b^ | +9.42 ^a,b^ | +9.99 ^a,b^ |  |
| Overall | 6.09  (5.30-6.89)^1^ | 11.23  (10.45-12.01)^1^ | 13.53  (12.10-14.96)^2^ | 13.32  (12.35-14.29)^1^ | 20.15  (17.81-22.49)^2^ | 20.34  (18.45-22.32)^2^ | 15.14  (12.91-17.36)^1^ | 22.67  (20.16-25.18)^1^ | 26.75  (23.96-29.54)^1^ | +7.23 ^a^ | +11.44 ^a,b^ | +13.22 ^a,b,c^ |  |
| ***Romania*** |  |  |  |  |  |  |  |  |  |  |  |  |  |
| Boys | 4.25  (3.15-5.35)^1^ | 8.80  (7.70-9.90)^1^ | 8.25  (7.15-9.35)^1^ | 7.48  (2.77-12.18)^2^ | 11.28  (6.19-16.38)^2^ | 12.21  (7.65-16.77)^2^ | - | - | - | +3.23 | +2.48 | +3.96 |  |
| Girls | 3.65  (2.55-4.75)^1^ | 10.95  (9.85-12.05)^1^ | 10.00  (8.90-11.10)^1^ | 6.01  (2.97-9.05)^2^ | 11.96  (5.94-17.99)^2(2)^ | 12.61  (5.90-19.32)^2^ | - | - | - | +2.36 | +1.01 | +2.60 |  |
| Overall | 3.95  (2.85-5.05)^1^ | 9.87  (8.77-10.97)^1^ | 9.12  (8.00-10.24)^1^ | 6.74  (2.87-10.61)^2^ | 11.66  (6.02-17.29)^2^ | 12.43  (6.84-18.02)^2^ | - | - | - | +2.79 | +1.79 | +3.31 |  |
| **Serbia** |  |  |  |  |  |  |  |  |  |  |  |  |  |
| Boys | 6.80  (5.30-8.60)^1^ | 11.50  (9.60-13.80)^1^ | 15.00  (12.90-17.40)^1^ | - | - | - | - | - | - | - | - | - |  |
| Girls | 4.10  (2.90-5.60)^1^ | 10.60  (8.90-12.60)^1^ | 16.40  (14.00-19.20)^1^ | - | - | - | - | - | - | - | - | - |  |
| Overall | 5.50  (4.50-6.70)^1^ | 11.90  (10.50-13.50)^1^ | 15.40  (13.80-17.10)^1^ | - | - | - | - | - | - | - | - | - |  |
| **Slovenia** |  |  |  |  |  |  |  |  |  |  |  |  |  |
| Boys | 6.30  (5.20-7.40)^1^ | 10.90  (9.90-11.90)^1^ | 12.00  (10.90-13.10)^1^ | 9.80  (8.70-10.90) ^1^ | 16.45  (15.45-17.45)^1^ | 15.70  (14.60-16.80)^1^ | - | - | - | +3.50 ^a^ | +5.55 ^a^ | +3.70 ^a^ |  |
| Girls | 7.05  (6.50-7.60)^1^ | 12.05  (10.95-13.15)^1^ | 12.35  (11.35-13.35)^1^ | 14.20  (13.20-15.20)^1^ | 18.10  (17.00-19.20)^1^ | 19.25  (18.15-20.35)^1^ | - | - | - | +7.15 ^a^ | +6.05 ^a^ | +6.90 ^a^ |  |
| Overall | 6.67  (5.57-7.77)^1^ | 11.47  (10.37-12.57)^1^ | 12.17  (11.07-13.27)^1^ | 12.00  (10.90-13.10)^1^ | 17.27  (16.17-18.37)^1^ | 17.47  (16.37-18.57)^1^ | - | - | - | +5.33 ^a^ | +5.80 ^a^ | +5.30 ^a^ |  |

|  | **1994-2003** | | | **2004-2012** | | | **2013-2022** | | | **Δ diabetes 1994-2022** | | |
| --- | --- | --- | --- | --- | --- | --- | --- | --- | --- | --- | --- | --- |
| Country | 0-4 yr | 5-9 yr | 10-14 yr | 0-4 yr | 5-9 yr | 10-14yr | 0-4 yr | 5-9 yr | 10-14 yr | 0-4 yr | 5-9 yr | 10-14 yr |
| ***Spain*** |  |  |  |  |  |  |  |  |  |  |  |  |
| Boys | 9.15  (5.32-12.98)^2^ | 14.73  (12.40-17.05)^2^ | 17.91  (13.69-22.14)^2^ | 10.19  (7.13-13.25)^2^ | 18.17  (13.69-22.65)^2^ | 21.40  (19.04-23.76)^2^ | 9.95  (5.90-14.00)^1^ | 20.66  (14.95-26.37)^1^ | 31.16  (23.89-38.43)^1^ | +0.8 | +5.93 | +13.25 ^b,c^ |
| Girls | 7.71  (5.12-10.29)^2^ | 15.33  (7.29-23.37)^2^ | 15.38  (12.78-17.97)^2^ | 9.01  (6.24-11.77)^2^ | 18.67  (13.99-23.34)^2^ | 18.05  (14.63-21.48)^2^ | 11.29  (6.80-15.78)^1^ | 21.02  (15.15-26.89)^1^ | 21.72  (15,59-27,85)^1^ | +3.58 | +5.69 | +6.34 |
| Overall | 7.81  (5.66-9.96)^2^ | 14.56  (13.74-15.38)^1^ | 17.72  (12.63-22.81)^2^ | 11.52  (7.18-15.86)^2^ | 21.97  (16.84-27.10)^2^ | 22.61  (18.16-27.06)^2^ | 14.15  (6.35-21.95)^2^ | 26.36  (14.81-37.91)^2^ | 31.17  (21.15-41.19)^2^ | +6.34 | +11.80 ^a^ | +13.45 |
| ***Sweden*** |  |  |  |  |  |  |  |  |  |  |  |  |
| Boys | 22.43  (20.71-24.15)^1^ | 32.39  (30.62-34.17)^1^ | 41.04  (39.23-42.85)^1^ | 25.16  (20.90-29.41)^2^ | 43.07  (37.31-48.84)^2^ | 55.96  (49.82-62.11)^2^ | - | - | - | +2.73 | +10.68 ^a^ | +14.92^a^ |
| Girls | 21.21  (19.53-22.89)^1^ | 34.94  (33.15-36.73)^1^ | 33.29  (29.00-37.58)^2^ | 23.41  (19.13-27.69)^2^ | 47.20  (37.68-56.71)^2^ | 45.94  (44.05-47.82)^1^ | - | - | - | +2.20 | +12.26 ^a^ | +12.65 ^a^ |
| Overall | 22.15  (20.62-23.68)^1^ | 33.98  (32.35-35.60)^1^ | 36.90  (35.24-38.56)^1^ | 24.76  (20.53-29.00)^2^ | 45.75  (38.14-53.36)^2^ | 50.94  (46.69-55.19)^2^ | - | - | - | +2.61 | +11.77 ^a^ | 14.03 ^a^ |
| ***Switzerland*** |  |  |  |  |  |  |  |  |  |  |  |  |
| Boys | 8.65  (7.75-9.55)^1^ | 9.90  (9.20-10.60)^1^ | 12.15  (11.45-12.85)^1^ | 10.60  (9.70-11.50)^1^ | 14.45  (13.75-15.15)^1^ | 15.80  (15.10-16.50)^1^ | - | - | - | +1.95 ^a^ | +4.55 ^a^ | +3.65 ^a^ |
| Girls | 6.55  (5.85-7.25)^1^ | 9.40  (8.70-10.10)^1^ | 11.05  (10.35-11.75)^1^ | 10.05  (9.35-10.75)^1^ | 13.95  (13.25-14.65)^1^ | 14.90  (14.20-15.60)^1^ | - | - | - | +3.50 ^a^ | +4.55 ^a^ | +3.85 |
| Overall | 8.30  (7.60-9.00)^1^ | 9.65  (8.95-10.35)^1^ | 11.60  (10.90-12.30)^1^ | 10.82  (10.12-11.52)^1^ | 14.20  (13.50-14.90)^1^ | 15.35  (14.65-16.05)^1^ | - | - | - | +2.52 ^a^ | +4.55 | +3.75 ^a^ |
| ***The Nethelands*** |  |  |  |  |  |  |  |  |  |  |  |  |
| Boys | - | - | - | 12.50  (10.40-15.00)^1^ | 22.10  (19.30-25.20)^1^ | 27.10  (24.00-30.50)^1^ | - | - | - | - | - | - |
| Girls | - | - | - | 12.30  (10.10-14.80)^1^ | 25.00  (22.00-28.40)^1^ | 28.00  (24.80-31.60)^1^ | - | - | - | - | - | - |
| Overall | 7.50  (2.00-13.00)^1^ | 26.00  (15.6-36.4)^1^ | 24.20  (21.90-26.60)^1^ | 12.40  (10.90-14.10)^1^ | 23.60  (21.50-25.80)^1^ | 27.60  (25.30-30.00)^1^ | - | - | - | +4.90 | -2.40 | +3.40 |

|  | **1994-2003** | | | **2004-2012** | | | **2013-2022** | | | **Δ diabetes 1994-2022** | | |
| --- | --- | --- | --- | --- | --- | --- | --- | --- | --- | --- | --- | --- |
| Country | 0-4 yr | 5-9 yr | 10-14 yr | 0-4 yr | 5-9 yr | 10-14yr | 0-4 yr | 5-9 yr | 10-14 yr | 0-4 yr | 5-9 yr | 10-14 yr |
| ***Turkey*** |  |  |  |  |  |  |  |  |  |  |  |  |
| Boys | - | - | - | 4.10  (0.08-8.12)^1^ | 6.20  (1.24-11.16)^1^ | 7.20  (1.87-12.53)^1^ | - | - | - | - | - | - |
| Girls | - | - | - | 4.40  (0.90-8.71)^1^ | 12.10  (4.95-19.25)^1^ | 9.60  (3.33-15.87)^1^ | - | - | - | - | - | - |
| Overall | - | - | - | 4.30  (1.32-7.28)^1^ | 9.10  (4.77-13.43)^1^ | 8.40  (4.28-12.52)^1^ | 7.61  (4.24-10.97)^2^ | 15.97  (7.14-24.80)^2^ | 15.33  (7.80-22.87)^2^ | +3.31 | +6.87 | +6.93 |
| ***United Kingdom*** |  |  |  |  |  |  |  |  |  |  |  |  |
| Boys | 14.93  (12.05-17.81)^2^ | 22.07  (19.40-24.73)^2^ | 31.86  (28.55-35.17)^2^ | 18.18  (14.91-21.45)^2^ | 28.88  (22.38-35.38)^2^ | 41.35  (32.40-50.30)^2^ | 14.20  (11.60-16.80)^1^ | 24.00  (20.60-27.40)^1^ | 28.60  (24.70-32.50)^1^ | -0.73 | +1.93 | -3.26 ^c^ |
| Girls | 14.94  (12.91-16.97)^2^ | 25.11  (21.96-28.27)^2^ | 26.87  (23.94-29.71)^2^ | 16.55  (12.95-20.15)^2^ | 32.47  (23.54-41.39)^2^ | 35.63  (29.74-41.72)^2^ | 13.40  (10.70-16.10)^1^ | 25.30  (21.70-28.90)^1^ | 21.60  (18.20-25.00)^1^ | -1.54 | +0.19 | -5.27 ^a,c^ |
| Overall | 15.93  (12.98-18.89)^2^ | 22.69  (20.16-25.21)^2^ | 28.76  (25.17-32.36)^2^ | 17.37  (13.99-20.74)^2^ | 30.67  (23.14-38.20)^2^ | 38.41  (31.39-45.43)^2^ | 13.80  (11.90-15.70)^1^ | 24.60  (22.10-27.10)^1^ | 25.20  (22.60-27.80)^1^ | -2.13 | +1.91 | -3.56^c^ |
| ^a^ Statistical significance between 1994-2012 period (p<0.05); ^b^ Statistical significance between 1994-2022 period (p<0.05); ^c^ Statistical significance between 2004-2022 period (p<0.05)  ^1^ Indicates the use of Fixed-Effect Model; ^2^ Indicates the use of Random-Effect Model | | | | | | | | | | | | |

# References of included studies

s1. Rami B, Waldhör T, Schober E. Incidence of Type I diabetes mellitus in children and young adults in the province of Upper Austria, 1994-1996. Diabetologia. 2001;44 Suppl 3:B45-7. doi:10.1007/pl00002953

s2. Schober E, Rami B, Waldhoer T. Steep increase of incidence of childhood diabetes since 1999 in Austria. Time trend analysis 1979-2005. A nationwide study. Eur J Pediatr. 2008;167(3):293-297. doi:10.1007/s00431-007-0480-5

s3. Bratina NU, Tahirović H, Battelino T, Krzisnik C. Incidence of childhood-onset Type I diabetes in Slovenia and the Tuzia region (Bosnia and Herzegovina) in the period 1990-1998. Diabetologia. 2001;44 Suppl 3:B27-31. doi:10.1007/pl00002949

s4. Bukara-Radujković G, Miljković V, Ljuboja O, Lakić S. Evidence for the increase in the incidence of type 1 diabetes in children aged 0-14 years in the Republika Srpska, 2001-2016. Cent Eur J Paediatr. 2018;14(1):61-67. doi:10.5457/p2005-114.200

s5. Stipancic G, La Grasta Sabolic L, Malenica M, Radica A, Skrabic V, Tiljak MK. Incidence and trends of childhood Type 1 diabetes in Croatia from 1995 to 2003. Diabetes Res Clin Pract. 2008;80(1):122-127. doi:10.1016/j.diabres.2007.10.019

s6. Rojnic Putarek N, Ille J, Spehar Uroic A, et al. Incidence of type 1 diabetes mellitus in 0 to 14-yr-old children in Croatia--2004 to 2012 study. Pediatr Diabetes. 2015;16(6):448-453. doi:10.1111/pedi.12197

s7. Mousa U, Sav H, Köseoğluları O, et al. The Incidence and Demographic Distribution of Type 1 Diabetes Mellitus in Children Aged 16 or Younger Between 2000 and 2016 in Cyprus. J Clin Res Pediatr Endocrinol. 2020;12(2):175-179. doi:10.4274/jcrpe.galenos.2019.2019.0109

s8. Toumba M, Savva SC, Bacopoulou I, et al. Rising incidence of type 1 diabetes mellitus in children and adolescents in Cyprus in 2000-2004. Pediatr Diabetes. 2007;8(6):374-376. doi:10.1111/j.1399-5448.2007.00262.x

s9. Cinek O, Lánská V, Kolousková S, et al. Type 1 diabetes mellitus in Czech children diagnosed in 1990-1997: a significant increase in incidence and male predominance in the age group 0-4 years. Collaborators of the Czech Childhood Diabetes Registry. Diabet Med. 2000;17(1):64-69. doi:10.1046/j.1464-5491.2000.00202.x

s10. Cinek O, Kulich M, Sumnik Z. The incidence of type 1 diabetes in young Czech children stopped rising. Pediatr Diabetes. 2012;13(7):559-563. doi:10.1111/j.1399-5448.2012.00858.x

s11. Teeäär T, Liivak N, Heilman K, et al. Increasing incidence of childhood-onset type 1 diabetes mellitus among Estonian children in 1999-2006. Time trend analysis 1983-2006. Pediatr Diabetes. 2010;11(2):107-110. doi:10.1111/j.1399-5448.2009.00535.

s12. Podar T, Solntsev A, Karvonen M, et al. Increasing incidence of childhood-onset type I diabetes in 3 Baltic countries and Finland 1983-1998. Diabetologia. 2001;44 Suppl 3:B17-20. doi:10.1007/pl00002947

s13. Rytkönen M, Moltchanova E, Ranta J, Taskinen O, Tuomilehto J, Karvonen M. The incidence of type 1 diabetes among children in Finland--rural-urban difference. Health Place. 2003;9(4):315-325. doi:10.1016/s1353-8292(02)00064-3

s14. Parviainen A, But A, Siljander H, Knip M. Decreased Incidence of Type 1 Diabetes in Young Finnish Children. Diabetes Care. 2020;43(12):2953-2958. doi:10.2337/dc20-0604

s15. Mauny F, Grandmottet M, Lestradet C, et al. Increasing trend of childhood type 1 diabetes in Franche-Comté (France): analysis of age and period effects from 1980 to 1998. Eur J Epidemiol. 2005;20(4):325-329. doi:10.1007/s10654-005-0329-z

s16. Barat P, Valade A, Brosselin P, Alberti C, Maurice-Tison S, Lévy-Marchal C. The growing incidence of type 1 diabetes in children: the 17-year French experience in Aquitaine. Diabetes Metab. 2008;34(6 Pt 1):601-605. doi:10.1016/j.diabet.2008.06.002

s17. Bendas A, Rothe U, Kiess W, et al. Trends in Incidence Rates during 1999-2008 and Prevalence in 2008 of Childhood Type 1 Diabetes Mellitus in Germany--Model-Based National Estimates. PLoS One. 2015;10(7):e0132716. doi:10.1371/journal.pone.0132716

s18. Ehehalt S, Blumenstock G, Willasch AM, Hub R, Ranke MB, Neu A. Continuous rise in incidence of childhood Type 1 diabetes in Germany. Diabet Med. 2008;25(6):755-757. doi:10.1111/j.1464-5491.2008.02450.x

s19. Galler A, Stange T, Müller G, et al. Incidence of childhood diabetes in children aged less than 15 years and its clinical and metabolic characteristics at the time of diagnosis: data from the Childhood Diabetes Registry of Saxony, Germany. Horm Res Paediatr. 2010;74(4):285-291. doi:10.1159/000303141

s20. Rosenbauer J, Icks A, Giani G. Incidence and prevalence of childhood type 1 diabetes mellitus in Germany--model-based national estimates. J Pediatr Endocrinol Metab. 2002;15(9):1497-1504. doi:10.1515/jpem.2002.15.9.1497

s21. Mamoulakis D, Vrouvaki F, Louvari V, Galanakis E. Incidence of childhood Type 1 diabetes mellitus in Crete. Diabet Med. May 2018. doi:10.1111/dme.13681

s22. Gyurus EK, Patterson C, Soltesz G. Twenty-one years of prospective incidence of childhood type 1 diabetes in Hungary--the rising trend continues (or peaks and highlands?). Pediatr Diabetes. 2012;13(1):21-25. doi:10.1111/j.1399-5448.2011.00826.x

s23. Bruno G, Maule M, Merletti F, et al. Age-period-cohort analysis of 1990-2003 incidence time trends of childhood diabetes in Italy: the RIDI study. Diabetes. 2010;59(9):2281-2287. doi:10.2337/db10-0151

s24. Fortunato F, Cappelli MG, Vece MM, et al. Incidence of Type 1 Diabetes among Children and Adolescents in Italy between 2009 and 2013: The Role of a Regional Childhood Diabetes Registry. J Diabetes Res. 2016;2016:7239692. doi:10.1155/2016/7239692

s25. Roche EF, Menon A, Gill D, Hoey HMC V. Incidence of type 1 diabetes mellitis in children aged under 15 years in the Republic of Ireland. J Pediatr Endocrinol Metab. 2002;15(8):1191-1194. doi:10.1515/jpem.2002.15.8.1191

s26. Roche EF, McKenna AM, Ryder KJ, Brennan AA, O’Regan M, Hoey HM. Is the incidence of type 1 diabetes in children and adolescents stabilising? The first 6 years of a National Register. Eur J Pediatr. 2016;175(12):1913-1919. doi:10.1007/s00431-016-2787-6

s27. Urbonaite B, Zalinkevicius R, Green A. Incidence, prevalence, and mortality of insulin-dependent (type 1) diabetes mellitus in Lithuanian children during 1983-98. Pediatr Diabetes. 2002;3(1):23-30. doi:10.1034/j.1399-5448.2002.30105.x

s28. Formosa N, Calleja N, Torpiano J. Incidence and modes of presentation of childhood type 1 diabetes mellitus in Malta between 2006 and 2010. Pediatr Diabetes. 2012;13(6):484-488. doi:10.1111/j.1399-5448.2011.00839.x

s29. Samardzic M, Marinkovic J, Kocev N, Curovic N, Terzic N. Increasing incidence of childhood type 1 diabetes in Montenegro from 1997 to 2006. Pediatr Diabetes. 2010;11(6):412-416. doi:10.1111/j.1399-5448.2009.00617.x

s30. Spaans EAJM, Gusdorf LMA, Groenier KH, et al. The incidence of type 1 diabetes is still increasing in the Netherlands, but has stabilised in children under five (Young DUDEs-1). Acta Paediatr. 2015;104(6):626-629. doi:10.1111/apa.12949

s31. Fazeli Farsani S, Souverein PC, van der Vorst MMJ, et al. Increasing trends in the incidence and prevalence rates of type 1 diabetes among children and adolescents in the Netherlands. Pediatr Diabetes. 2016;17(1):44-52. doi:10.1111/pedi.12232

s32. Aamodt G, Stene LC, Njølstad PR, Søvik O, Joner G. Spatiotemporal trends and age-period-cohort modeling of the incidence of type 1 diabetes among children aged <15 years in Norway 1973-1982 and 1989-2003. Diabetes Care. 2007;30(4):884-889. doi:10.2337/dc06-1568

s33. Skrivarhaug T, Stene LC, Drivvoll AK, Strøm H, Joner G. Incidence of type 1 diabetes in Norway among children aged 0-14 years between 1989 and 2012: has the incidence stopped rising? Results from the Norwegian Childhood Diabetes Registry. Diabetologia. 2014;57(1):57-62. doi:10.1007/s00125-013-3090-y

s34. Jarosz-Chobot P, Polanska J, Szadkowska A, et al. Rapid increase in the incidence of type 1 diabetes in Polish children from 1989 to 2004, and predictions for 2010 to 2025. Diabetologia. 2011;54(3):508-515. doi:10.1007/s00125-010-1993-4

s35. Szalecki M, Wysocka-Mincewicz M, Ramotowska A, et al. Epidemiology of type 1 diabetes in Polish children: A multicentre cohort study. Diabetes Metab Res Rev. 2018;34(2). doi:10.1002/dmrr.2962

s36. Chobot A, Polanska J, Brandt A, et al. Updated 24-year trend of Type 1 diabetes incidence in children in Poland reveals a sinusoidal pattern and sustained increase. Diabet Med. 2017;34(9):1252-1258. doi:10.1111/dme.13345

s37. Serban V, Brink S, Timar B, et al. An increasing incidence of type 1 diabetes mellitus in Romanian children aged 0 to 17 years. J Pediatr Endocrinol Metab. 2015;28(3-4):293-298. doi:10.1515/jpem-2014-0364

s38. Vlad A, Serban V, Green A, et al. Time Trends, Regional Variability and Seasonality Regarding the Incidence of Type 1 Diabetes Mellitus in Romanian Children Aged 0-14 Years, Between 1996 and 2015. J Clin Res Pediatr Endocrinol. 2018;10(2):92-99. doi:10.4274/jcrpe.5456

s39. Sipetic S, Maksimovic J, Vlajinac H, et al. Rising incidence of type 1 diabetes in Belgrade children aged 0-14 years in the period from 1982 to 2005. J Endocrinol Invest. 2013;36(5):307-312. doi:10.3275/8619

s40. Bahíllo MP, Hermoso F, Ochoa C, et al. Incidence and prevalence of type 1 diabetes in children aged <15 yr in Castilla-Leon (Spain). Pediatr Diabetes. 2007;8(6):369-373. doi:10.1111/j.1399-5448.2007.00255.x

s41. Fernández-Ramos C, Arana-Arri E, Jiménez-Huertas P, Vela A, Rica I. Incidence of childhood-onset type 1 diabetes in Biscay, Spain, 1990-2013. Pediatr Diabetes. 2017;18(1):71-76. doi:10.1111/pedi.12354

s42. Forga L, Chueca MJ, Tamayo I, Oyarzabal M, Toni M, Goñi MJ. Cyclical variation in the incidence of childhood-onset type 1 diabetes during 40 years in Navarra (Spain). Pediatr Diabetes. 2018;19(8):1416-1421. doi:10.1111/pedi.12758

s43. Gimeno Benítez A, Luengo Pérez LM, Suero Villa P, Suero Villa S, Sánchez Vega J. [Incidence of childhood type I diabetes in Extremadura, Spain, 2003-2007]. Semergen. 2014;40(4):177-182. doi:10.1016/j.semerg.2013.10.005

s44. Muiña PG, Herrera MJB, Atance EP, Donado JJA, Sánchez G, Ferrer LS. [Epidemiological study of type 1 diabetes in children under 15 years-old in Castilla-La Mancha (Spain)]. An Pediatr (Barc). 2012;76(2):83-91. doi:10.1016/j.anpedi.2011.02.007

s45. López-Siguero JP, Del Pino-De la Fuente A, Martínez-Aedo MJ, Moreno-Molina JA. Increased incidence of type 1 diabetes in the south of Spain. Diabetes Care. 2002;25(6):1099. doi:10.2337/diacare.25.6.1099

s46. Lora-Gómez RE, Morales-Pérez FM, Arroyo-Díez FJ, Barquero-Romero J. Incidence of Type 1 diabetes in children in Cáceres, Spain, during 1988-1999. Diabetes Res Clin Pract. 2005;69(2):169-174. doi:10.1016/j.diabres.2004.11.013

s47. Morales-Pérez FM, Barquero-Romero J, Pérez-Miranda M. Incidence of type I diabetes among children and young adults (0-29 years) in the province of Badajoz, Spain during 1992 to 1996. Acta Paediatr. 2000;89(1):101-104. doi:10.1080/080352500750029158

s48. Mayoral González B, Riaño Galán I, Rodriguez Dehli C, Labra Alvarez R, Díaz Naya L, Menéndez Torre E. Epidemiology of type 1 diabetes in Asturias: 2002-2011. Endocrinol diabetes y Nutr. 2018;65(2):68-73. doi:10.1016/j.endinu.2017.10.013

s49. Nóvoa Y, de La Cuesta A, Caballero E, et al. Epidemiology of childhood-onset type 1 diabetes in Gran Canaria (2006-2018). Endocrinol diabetes y Nutr. 2020;67(10):658-664. doi:10.1016/j.endinu.2019.11.010

s50. Soria J, Garagorri JM, Rodríguez M, Rodríguez G, Larrad L, Elizalde M. Epidemiology and genetic risk of type 1 diabetes among children in Aragon community, Spain. Diabetes Res Clin Pract. 2008;79(1):112-116. doi:10.1016/j.diabres.2007.06.013

s51. Zorrilla Torras B, Cantero Real JL, Barrios Castellanos R, Ramírez Fernández J, Argente Oliver J, González Vergaz A. [Incidence of type 1 diabetes mellitus in children: results from the population registry of the Madrid Region, 1997-2005]. Med Clin (Barc). 2009;132(14):545-548. doi:10.1016/j.medcli.2008.05.020

s52. Berhan Y, Waernbaum I, Lind T, Möllsten A, Dahlquist G. Thirty years of prospective nationwide incidence of childhood type 1 diabetes: the accelerating increase by time tends to level off in Sweden. Diabetes. 2011;60(2):577-581. doi:10.2337/db10-0813

s53. Demirbilek H, Özbek MN, Baran RT. Incidence of type 1 diabetes mellitus in Turkish children from the southeastern region of the country: a regional report. J Clin Res Pediatr Endocrinol. 2013;5(2):98-103. doi:10.4274/Jcrpe.954

s54. Esen I, Okdemir D. Trend of type 1 diabetes incidence in children between 2009 and 2019 in Elazig, Turkey. Pediatr Diabetes. 2020;21(3):460-465. doi:10.1111/pedi.12984

s55. Poyrazoğlu Ş, Bundak R, Yavaş Abalı Z, et al. Incidence of Type 1 Diabetes in Children Aged Below 18 Years during 2013-2015 in Northwest Turkey. J Clin Res Pediatr Endocrinol. 2018;10(4):336-342. doi:10.4274/jcrpe.0025

s56. Zhao HX, Stenhouse E, Soper C, et al. Incidence of childhood-onset Type 1 diabetes mellitus in Devon and Cornwall, England, 1975-1996. Diabet Med. 1999;16(12):1030-1035. doi:10.1046/j.1464-5491.1999.00175.x

s57. Harron KL, McKinney PA, Feltbower RG, et al. Incidence rate trends in childhood type 1 diabetes in Yorkshire, UK 1978-2007: effects of deprivation and age at diagnosis in the South Asian and non-South Asian populations. Diabet Med. 2011;28(12):1508-1513. doi:10.1111/j.1464-5491.2011.03413.x

s58. Raymond NT, Jones JR, Swift PG, et al. Comparative incidence of Type I diabetes in children aged under 15 years from South Asian and White or Other ethnic backgrounds in Leicestershire, UK, 1989 to 1998. Diabetologia. 2001;44 Suppl 3:B32-6. doi:10.1007/pl00002951

s59. Cardwell CR, Carson DJ, Patterson CC. Higher incidence of childhood-onset type 1 diabetes mellitus in remote areas: a UK regional small-area analysis. Diabetologia. 2006;49(9):2074-2077. doi:10.1007/s00125-006-0342-0

s60. Knip M, Parviainen A, Turtinen M, But A, Härkönen T, Hepojoki J, Sironen T, Iheozor-Ejiofor R, Uğurlu H, Saksela K, Lempainen J, Ilonen J, Vapalahti O; Finnish Pediatric Diabetes Register. SARS-CoV-2 and type 1 diabetes in children in Finland: an observational study. Lancet Diabetes Endocrinol. 2023 Apr;11(4):251-260. Doi: 10.1016/S2213-8587(23)00041-4.

s61. Rodríguez Escobedo R, Delgado Álvarez E, Menéndez Torre EL. Incidence of type 1 diabetes mellitus in Asturias (Spain) between 2011 and 2020. Endocrinol Diabetes Nutr (Engl Ed). 2023 Mar;70(3):189-195. Doi: 10.1016/j.endien.2023.03.008

s62. Hayes L, Cheetham T, Muirhead C, Hopper N, Reid J, Lamb W, Foster J, McNally RJQ. Type 1 diabetes in North East England and North Cumbria: patterns and time trends in 0-14-year-olds from 2012 to 2020. Front Public Health. 2023 Aug 11;11:1193403. Doi: 10.3389/fpubh.2023.1193403

s63. Baechle C, Eckert A, Kamrath C, Neu A, Manuwald U, Thiele-Schmitz S, Weidler O, Knauer-Fischer S, Rosenbauer J, Holl RW. Incidence and presentation of new-onset type 1 diabetes in children and adolescents from Germany during the COVID-19 pandemic 2020 and 2021: Current data from the DPV Registry. Diabetes Res Clin Pract. 2023 Mar;197:110559. Doi: 10.1016/j.diabres.2023.110559.

s64. Hernández Herrero M, Terradas Mercader P, Latorre Martinez E, Feliu Rovira A, Rodríguez Zaragoza N, Parada Ricart E. New diagnoses of type 1 diabetes mellitus in children during the COVID-19 pandemic Regional multicenter study in Spain. Endocrinol Diabetes Nutr (Engl Ed). 2022 Nov;69(9):709-714. Doi: 10.1016/j.endien.2021.12.009.

s65. Roche EF, McKenna AM, O’Regan M, Ryder KJ, Fitzgerald HM, Hoey HMCV. The incidence of type 1 diabetes in children under 15 years of age is rising again-a nationwide study. Eur J Pediatr. 2023 Oct;182(10):4615-4623. Doi: 10.1007/s00431-023-05125-7.

s66. Weiss A, Donnachie E, Beyerlein A, Ziegler AG, Bonifacio E. Type 1 Diabetes Incidence and Risk in Children With a Diagnosis of COVID-19. JAMA. 2023 Jun 20;329(23):2089-2091. doi: 10.1001/jama.2023.8674.

s67. Gesuita R, Rabbone I, Marconi V, De Sanctis L, Marino M, Tiberi V, Iannilli A, Tinti D, Favella L, Giorda C, Carle F, Cherubini V. Trends and cyclic variation in the incidence of childhood type 1 diabetes in two Italian regions over 33 years and during the COVID-19 pandemic. Diabetes Obes Metab. 2023 Jun;25(6):1698-1703. Doi: 10.1111/dom.15024.

s68. Raicevic M, Samardzic M, Soldatovic I, Curovic Popovic N, Vukovic R. Trends in nationwide incidence of pediatric type 1 diabetes in Montenegro during the last 30 years. Front Endocrinol (Lausanne). 2022 Sep 6;13:991533. Doi: 10.3389/fendo.2022.991533.

s69. Vorgučin I, Savin M, Stanković Đ, Miljković D, Ilić T, Simić D, Vrebalov M, Milanović B, Barišić N, Stojanović V, Vijatov-Đurić G, Koprivšek K, Vilotijević-Dautović G, Antić J. Incidence of Type 1 Diabetes Mellitus and Characteristics of Diabetic Ketoacidosis in Children and Adolescents during the First Two Years of the COVID-19 Pandemic in Vojvodina. Medicina (Kaunas). 2022 Jul 28;58(8):1013. Doi: 10.3390/medicina58081013.

s70. Cinek O, Slavenko M, Pomahačová R, Venháčová P, Petruželková L, Škvor J, Neumann D, Vosáhlo J, Konečná P, Kocourková K, Strnadel J, Průhová Š, Šumník Z; ČENDA Register. Type 1 diabetes incidence increased during the COVID-19 pandemic years 2020-2021 in Czechia: Results from a large population-based pediatric register. Pediatr Diabetes. 2022 Nov;23(7):956-960. Doi: 10.1111/pedi.13405.

s71. Giorda CB, Gnavi R, Tartaglino B, Manti R, Migliardi A, Favella L, Ferro S, Rabbone I. Increased incidence of type 1 diabetes in 2 years of COVID-19 pandemic. Acta Diabetol. 2023 Apr;60(4):587-589. Doi: 10.1007/s00592-022-01986-w.

s72. Passanisi S, Salzano G, Aloe M, Bombaci B, Citriniti F, De Berardinis F, De Marco R, Lazzaro N, Lia MC, Lia R, Mammì F, Stamati FA, Toscano RMR, Ventrici C, Iafusco D, Lombardo F. Increasing trend of type 1 diabetes incidence in the pediatric population of the Calabria region in 2019-2021. Ital J Pediatr. 2022 May 4;48(1):66. doi: 10.1186/s13052-022-01264-z.

s73. Goñi Iriarte MJ, Brugos Larumbe A, Guillén Grima F, Sainz de Los Terreros Errea A, Chueca Guendulain MJ, Forga Llenas L. Incidence of type 1 diabetes in Navarra, 2009-2020. Evidence of a stabilization. Endocrinol Diabetes Nutr (Engl Ed). 2023 Feb;70(2):80-87. doi: 10.1016/j.endien.2023.02.005

# PRISMA Checklist

| **Section and Topic** | **Item #** | **Checklist item** | **Location where item is reported** |
| --- | --- | --- | --- |
| **TITLE** | | |  |
| Title | 1 | Identify the report as a systematic review. | Page 1 |
| **ABSTRACT** | | |  |
| Abstract | 2 | See the PRISMA 2020 for Abstracts checklist. | 2 |
| **INTRODUCTION** | | |  |
| Rationale | 3 | Describe the rationale for the review in the context of existing knowledge. | 4-5 |
| Objectives | 4 | Provide an explicit statement of the objective(s) or question(s) the review addresses. | 4-5 |
| **METHODS** | | |  |
| Eligibility criteria | 5 | Specify the inclusion and exclusion criteria for the review and how studies were grouped for the syntheses. | 5-6 |
| Information sources | 6 | Specify all databases, registers, websites, organisations, reference lists and other sources searched or consulted to identify studies. Specify the date when each source was last searched or consulted. | 5-6 |
| Search strategy | 7 | Present the full search strategies for all databases, registers and websites, including any filters and limits used. | 5 and Sup table 1 |
| Selection process | 8 | Specify the methods used to decide whether a study met the inclusion criteria of the review, including how many reviewers screened each record and each report retrieved, whether they worked independently, and if applicable, details of automation tools used in the process. | 5-7 |
| Data collection process | 9 | Specify the methods used to collect data from reports, including how many reviewers collected data from each report, whether they worked independently, any processes for obtaining or confirming data from study investigators, and if applicable, details of automation tools used in the process. | 5-7 |
| Data items | 10a | List and define all outcomes for which data were sought. Specify whether all results that were compatible with each outcome domain in each study were sought (e.g. for all measures, time points, analyses), and if not, the methods used to decide which results to collect. | 6-7 |
|  | 10b | List and define all other variables for which data were sought (e.g. participant and intervention characteristics, funding sources). Describe any assumptions made about any missing or unclear information. | 6-8 |
| Study risk of bias assessment | 11 | Specify the methods used to assess risk of bias in the included studies, including details of the tool(s) used, how many reviewers assessed each study and whether they worked independently, and if applicable, details of automation tools used in the process. | 7 |
| Effect measures | 12 | Specify for each outcome the effect measure(s) (e.g. risk ratio, mean difference) used in the synthesis or presentation of results. | 7-8 |
| Synthesis methods | 13a | Describe the processes used to decide which studies were eligible for each synthesis (e.g. tabulating the study intervention characteristics and comparing against the planned groups for each synthesis (item #5)). | 7-8 |
|  | 13b | Describe any methods required to prepare the data for presentation or synthesis, such as handling of missing summary statistics, or data conversions. | 7-8 |
|  | 13c | Describe any methods used to tabulate or visually display results of individual studies and syntheses. | 7-8 |
|  | 13d | Describe any methods used to synthesize results and provide a rationale for the choice(s). If meta-analysis was performed, describe the model(s), method(s) to identify the presence and extent of statistical heterogeneity, and software package(s) used. | 7-8 |
|  | 13e | Describe any methods used to explore possible causes of heterogeneity among study results (e.g. subgroup analysis, meta-regression). | 7-8 |
|  | 13f | Describe any sensitivity analyses conducted to assess robustness of the synthesized results. | 7-8 |
| Reporting bias assessment | 14 | Describe any methods used to assess risk of bias due to missing results in a synthesis (arising from reporting biases). | 7 |
| Certainty assessment | 15 | Describe any methods used to assess certainty (or confidence) in the body of evidence for an outcome. | 7 |
| **RESULTS** | | |  |
| Study selection | 16a | Describe the results of the search and selection process, from the number of records identified in the search to the number of studies included in the review, ideally using a flow diagram. | 8, 9 and figure 1 |
|  | 16b | Cite studies that might appear to meet the inclusion criteria, but which were excluded, and explain why they were excluded. | Flow diagram |
| Study characteristics | 17 | Cite each included study and present its characteristics. | 8,9, Suppl table 2 and supp material |
| Risk of bias in studies | 18 | Present assessments of risk of bias for each included study. | 9-10 and Suppl table 3 |
| Results of individual studies | 19 | For all outcomes, present, for each study: (a) summary statistics for each group (where appropriate) and (b) an effect estimate and its precision (e.g. confidence/credible interval), ideally using structured tables or plots. | 9-11, table 1, table suppl 4 |
| Results of syntheses | 20a | For each synthesis, briefly summarise the characteristics and risk of bias among contributing studies. | 9-11 |
|  | 20b | Present results of all statistical syntheses conducted. If meta-analysis was done, present for each the summary estimate and its precision (e.g. confidence/credible interval) and measures of statistical heterogeneity. If comparing groups, describe the direction of the effect. | 9-11 and figure 2 |
|  | 20c | Present results of all investigations of possible causes of heterogeneity among study results. | figure 3 |
|  | 20d | Present results of all sensitivity analyses conducted to assess the robustness of the synthesized results. | 16 and figure 4 |
| Reporting biases | 21 | Present assessments of risk of bias due to missing results (arising from reporting biases) for each synthesis assessed. | Suppl table 2 |
| Certainty of evidence | 22 | Present assessments of certainty (or confidence) in the body of evidence for each outcome assessed. | Table 1, fig 3, suppl table 3 |
| **DISCUSSION** | | |  |
| Discussion | 23a | Provide a general interpretation of the results in the context of other evidence. | 17-23 |
|  | 23b | Discuss any limitations of the evidence included in the review. | 22 |
|  | 23c | Discuss any limitations of the review processes used. | 22 |
|  | 23d | Discuss implications of the results for practice, policy, and future research. | 17-23 |
| **OTHER INFORMATION** | | |  |
| Registration and protocol | 24a | Provide registration information for the review, including register name and registration number, or state that the review was not registered. | 5 |
|  | 24b | Indicate where the review protocol can be accessed, or state that a protocol was not prepared. | PROSPERO registration |
|  | 24c | Describe and explain any amendments to information provided at registration or in the protocol. |  |
| Support | 25 | Describe sources of financial or non-financial support for the review, and the role of the funders or sponsors in the review. | 23 |
| Competing interests | 26 | Declare any competing interests of review authors. | 23 |
| Availability of data, code and other materials | 27 | Report which of the following are publicly available and where they can be found: template data collection forms; data extracted from included studies; data used for all analyses; analytic code; any other materials used in the review. | 23, Under request to CA |

*From:*  Page MJ, McKenzie JE, Bossuyt PM, Boutron I, Hoffmann TC, Mulrow CD, et al. The PRISMA 2020 statement: an updated guideline for reporting systematic reviews. BMJ 2021;372:n71. doi: 10.1136/bmj.n71

For more information, visit: <http://www.prisma-statement.org/>
